# Supplementary material for: Isostructural doping for organic persistent mechanoluminescence
Source: Nat Commun. 2024 Apr 30;15:3668. doi: 10.1038/s41467-024-47962-6 (PMC11063035; doi:10.1038/s41467-024-47962-6)
Supplement: Supplementary file 1 — Supplementary Information [file 41467_2024_47962_MOESM1_ESM.pdf]

Supplementary Information

Isostructural doping for Organic Persistent Mechanoluminescence

Zongliang Xie[1,2], Yufeng Xue[2], Xianhe Zhang[2], Junru Chen[2], Zesen Lin[1,2] and Bin Liu*[1,2]

[1] Institute for Functional Intelligent Materials, National University of Singapore, Singapore, Singapore.

[2] Department of Chemical and Biomolecular Engineering, National University of Singapore, Singapore, Singapore.

*E-mail: cheliub@nus.edu.sg

CONTENT

SUPPLEMENTARY METHODS.......... 3
SUPPLEMENTARY FIGURES.......... 8
SUPPLEMENTARY TABLES.......... 40
SUPPLEMENTARY REFERENCES.......... 44

Supplementary Methods

Materials and syntheses

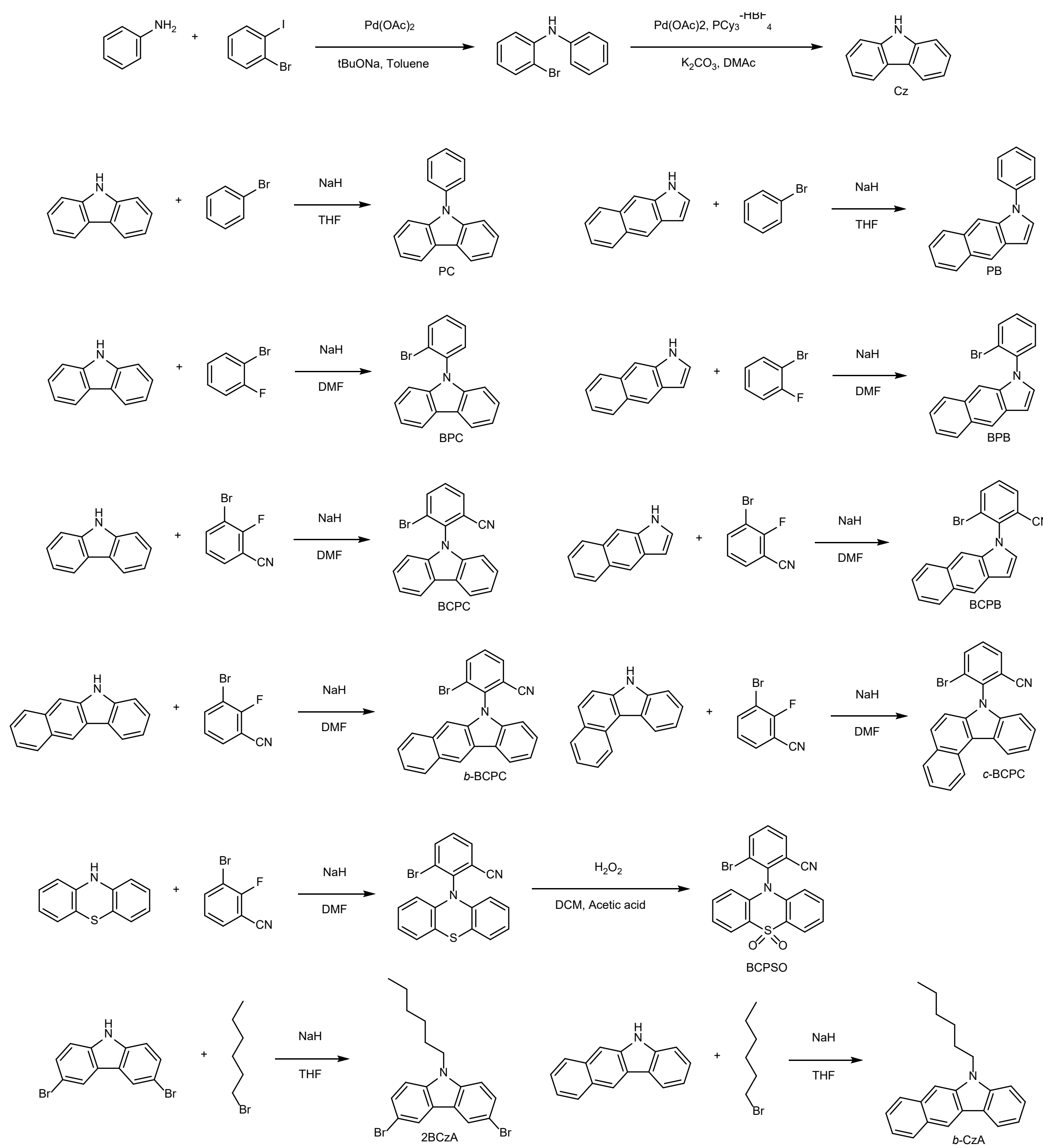

Supplementary Fig. 1 | Synthetic routes for target compounds.

Synthetic routes for relative isostructural host and guest molecules are described as Supplementary Fig. 1. Detailed synthetic methods are described as follows. All the products were purified by column chromatography and were confirmed by ^{1}H NMR, ^{13}C NMR, and EI-HRMS.

Synthesis of 9-phenyl-9H-carbazole (PC)

9H-carbazole (0.80 g, 4.78 mmol) was dissolved in anhydrous tetrahydrofuran (THF) (30 mL). Then sodium hydride (0.57 g, 14.35 mmol) dispersed in 60% mineral oil was added. After stirring for 15 min under N_2 atmosphere, bromobenzene (0.90 g, 5.74 mmol) was added to the solution. The reaction mixture was stirred at 80 °C for 6 h, and then 100 mL of water was added to quench the reaction. The resulting mixture was extracted with dichloromethane three times. The separated organic layer was evaporated under reduced pressure to yield the crude product as a light-grey powder. Chromatography on a silica-gel column with dichloromethane-hexane (1/5, v/v) as eluent was then performed for the further purification to yield as a white powder (0.89 g, 3.66 mmol) with 76.5% yield. ^{1}H NMR (400 MHz, $CDCl_3$) δ (TMS, ppm) 8.16 (d, 2H), 7.65 – 7.56 (m, 4H), 7.50 – 7.45 (m, 1H), 7.42 (d, 4H), 7.30 (p, 2H). ^{13}C NMR (101 MHz, $CDCl_3$) δ 141.00, 137.81, 130.03, 127.58, 127.27, 126.04, 123.45, 120.43, 120.01, 109.89. EI-HRMS: Meas. m/z: 243.1041; Calc. Mass for $C_{18}H_{13}N$: 243.1043.

Synthesis of 1-phenyl-1H-benzo[f]indole (PB)

PB was synthesized according to the synthetic procedure of PC with 1H-benzo[f]indole (50.0 mg, 299.0 mmol), sodium hydride (35.9 mg, 897.1 mmol) dispersed in 60% mineral oil, bromobenzene (56.3 mg, 358.8 mmol), and 10 mL of anhydrous THF. The crude product was purified by Chromatography on a silica-gel column with dichloromethane-hexane (1/5, v/v) as eluent to afford the target product of PB as white powder (41.6 mg, 171.0 mmol) with 57.2% yield. ^{1}H NMR (400 MHz, $CDCl_3$) δ (TMS, ppm): 8.18 (s, 1H), 8.03 (s, 1H), 7.99 – 7.93 (m, 1H), 7.90 – 7.84 (m, 1H), 7.64 – 7.54 (m, 5H), 7.42 – 7.33 (m, 3H), 6.82 (d, 1H). ^{13}C NMR (101 MHz, $CDCl_3$) δ 139.86, 136.12, 131.59, 130.54, 130.23, 129.58, 128.75, 127.90, 127.43, 126.07, 123.98, 123.79, 122.80, 118.37, 105.76, 102.91. EI-HRMS: Meas. m/z: 243.1041; Calc. Mass for $C_{18}H_{13}N$: 243.1043.

Synthesis of 9-(2-bromophenyl)-9H-carbazole (BPC)

9H-carbazole (0.80 g, 4.78 mmol) was dissolved in 30 mL of anhydrous N, N-dimethyl formamide (DMF). Then sodium hydride (0.23 g, 5.74 mmol) dispersed in 60% mineral oil was added to the solution at 0 °C. After stirring for 30 minutes under N_2 atmosphere, 1-bromo-2-fluorobenzene (1.00 g, 5.74 mmol) was added. Then the reaction mixture was heated to reflux and stirred for 12 hours. After cooling to room temperature, the reaction mixture was slowly poured into 300 mL of ice water while stirred. The mixture

was then filtered to obtain crude product as light-yellow powders. Chromatography on a silica-gel column with dichloromethane-hexane (1/5, v/v) as eluent was then performed for further purification to yield BPC as a white powder (1.07 g, 3.32 mmol) with 69.4% yield. ^{1}H NMR (400 MHz, $CDCl_3$) δ (TMS, ppm) 8.16 (d, 2H), 7.87 (dd, 1H), 7.51 (m, 2H), 7.41 (q, 3H), 7.30 (t, 2H), 7.07 (d, 2H). ^{13}C NMR (101 MHz, $CDCl_3$) δ 140.99, 136.90, 134.39, 131.29, 130.30, 128.96, 126.08, 124.00, 123.39, 120.49, 120.16, 110.20. EI-HRMS: Meas. m/z: 321.1046; Calc. Mass for $C_{18}H_{12}BrN$: 321.1048.

Synthesis of 1-(2-bromophenyl)-1H-benzo[f]indole (BPB)

BPB was synthesized according to the synthetic procedure of BPC with 1H-benzo[f]indole (50.0 mg, 299.0 mmol), sodium hydride (14.4 mg, 358.8 mmol) dispersed in 60% mineral oil, 1-bromo-2-fluorobenzene (62.8 mg, 358.8 mmol), and 10 mL DMF. The crude product was purified by Chromatography on a silica-gel column with dichloromethane-hexane (1/5, v/v) as eluent to afford the target product of BPB as white powder (47.4 mg, 147.1 mmol) with 49.2% yield. ^{1}H NMR (400 MHz, $CDCl_3$) δ (TMS, ppm): 8.17 (s, 1H), 7.96 (d, 2H), 7.86 (d, 1H), 7.72 – 7.66 (m, 2H), 7.52 – 7.47 (m, 3H), 7.40 – 7.34 (m, 2H), 6.82 (s, 1H). ^{13}C NMR (101 MHz, $CDCl_3$) δ 138.81, 137.48, 134.29, 132.73, 130.55, 130.04, 130.00, 129.66, 129.13, 128.64, 128.30, 127.60, 124.11, 123.04, 122.28, 118.62, 106.11, 102.77. EI-HRMS: Meas. m/z: 321.1049; Calc. Mass for $C_{18}H_{12}BrN$: 321.1048.

Synthesis of 3-bromo-2-(9H-carbazol-9-yl)benzonitrile (BCPC)

BCPC was synthesized according to the synthetic procedure of BPC with 9H-carbazole (2.00 g, 11.96 mmol), sodium hydride (0.57 g, 14.35 mmol) dispersed in 60% mineral oil, 3-bromo-2-fluorobenzonitrile (2.87 g, 14.35 mmol), and 40 mL of DMF. The crude product was purified by Chromatography on a silica-gel column with dichloromethane-hexane (1/3, v/v) as eluent to afford the target product of BCPC as white crystalline powders (3.31 g, 9.53 mmol) with 79.7% yield. ^{1}H NMR (400 MHz, $CDCl_3$) δ (TMS, ppm): 8.17 (d, 2H), 8.09 (d, 1H), 7.89 (d, 1H), 7.55 (t, 1H), 7.43 (t, 2H), 7.34 (t, 2H), 6.99 (d, 2H). ^{13}C NMR (101 MHz, $CDCl_3$) δ 140.16, 139.55, 138.81, 133.35, 130.60, 126.47, 126.13, 123.99, 121.02, 120.87, 116.63, 115.01, 109.69. EI-HRMS: Meas. m/z: 346.0100; Calc. Mass for $C_{19}H_{11}BrN_2$: 346.0100.

Synthesis of 2-(1H-benzo[f]indol-1-yl)-3-bromobenzonitrile (BCPB)

BCPB was synthesized according to the synthetic procedure of BPC with 1H-benzo[f]indole (50.0 mg, 299.0 mmol), sodium hydride (14.4 mg, 358.8 mmol) dispersed in 60% mineral oil, 3-bromo-2-fluorobenzonitrile

(71.8 mg, 358.8 mmol), and 10 mL of DMF. The crude product was purified by Chromatography on a silica-gel column with dichloromethane-hexane (1/3, v/v) as eluent to afford the target product of BCPB as light-yellow powders (66.2 mg, 190.7 mmol) with 63.8% yield. ^{1}H NMR (400 MHz, $CDCl_3$) δ (TMS, ppm): 8.21 (s, 1H), 8.06 (d, 1H), 7.98 (dd, 1H), 7.87 – 7.80 (m, 2H), 7.51 (t, 1H), 7.40 – 7.34 (m, 4H), 6.93 (d, 1H). ^{13}C NMR (101 MHz, $CDCl_3$) δ 141.28, 138.58, 136.79, 133.12, 131.40, 130.82, 130.20, 129.92, 129.51, 128.42, 127.61, 125.15, 124.48, 123.39, 119.30, 115.64, 115.14, 105.97, 104.85. EI-HRMS: Meas. m/z: 346.0104; Calc. Mass for $C_{19}H_{11}BrN_2$: 346.0100.

Synthesis of 2-(5H-benzo[b]carbazol-5-yl)-3-bromobenzonitrile (*b*-BCPC)

b-BCPC was synthesized according to the synthetic procedure of BPC with 5H-benzo[b]carbazole (50.0 mg, 230.1 mmol), sodium hydride (11.1 mg, 276.2 mmol) dispersed in 60% mineral oil, 3-bromo-2-fluorobenzonitrile (55.2 mg, 276.2 mmol), and 10 mL of DMF. The crude product was purified by Chromatography on a silica-gel column with dichloromethane-hexane (1/3, v/v) as eluent to afford the target product of *b*-BCPC as white powders (39.6 mg, 99.7 mmol) with 43.3% yield. ^{1}H NMR (400 MHz, $CDCl_3$) δ (TMS, ppm): 8.63 (s, 1H), 8.28 (d, *J* = 7.7 Hz, 1H), 8.16 – 8.07 (m, 2H), 7.94 (dd, *J* = 7.8, 1.5 Hz, 1H), 7.86 – 7.82 (m, 1H), 7.59 (t, *J* = 8.0 Hz, 1H), 7.50 – 7.42 (m, 3H), 7.36 (t, *J* = 7.1 Hz, 1H), 7.28 (s, 1H), 6.96 (d, *J* = 8.1 Hz, 1H). ^{13}C NMR (101 MHz, $CDCl_3$) δ 142.47, 139.94, 139.69, 139.02, 133.55, 132.88, 130.71, 129.33, 128.70, 127.80, 127.37, 126.35, 125.63, 123.86, 123.61, 121.55, 121.12, 119.45, 116.93, 115.06, 109.38, 104.80. EI-HRMS: Meas. m/z: 396.0252; Calc. Mass for $C_{23}H_{13}BrN_2$: 396.0257.

Synthesis of 2-(7H-benzo[c]carbazol-7-yl)-3-bromobenzonitrile (*c*-BCPC)

c-BCPC was synthesized according to the synthetic procedure of BPC with 7H-benzo[c]carbazole (50.0 mg, 230.1 mmol), sodium hydride (11.1 mg, 276.2 mmol) dispersed in 60% mineral oil, 3-bromo-2-fluorobenzonitrile (55.2 mg, 276.2 mmol), and 10 mL of DMF. The crude product was purified by Chromatography on a silica-gel column with dichloromethane-hexane (1/3, v/v) as eluent to afford the target product of *c*-BCPC as white powders (44.1 mg, 111.0 mmol) with 48.2% yield. ^{1}H NMR (400 MHz, $CDCl_3$) δ (TMS, ppm): 8.86 (d, 1H), 8.69 – 8.63 (m, 1H), 8.12 (m, 1H), 8.03 (d, 1H), 7.95 – 7.86 (m, 2H), 7.79 – 7.72 (m, 1H), 7.63 – 7.57 (m, 1H), 7.55 – 7.44 (m, 3H), 7.17 (dt, 1H), 7.13 – 7.06 (m, 1H). ^{13}C NMR (101 MHz, $CDCl_3$) δ 139.35, 138.78, 137.89, 133.33, 130.87, 130.05, 130.01, 129.44, 128.12, 127.34, 126.37,

125.14, 124.65, 123.74, 123.69, 122.66, 121.72, 116.79, 116.66, 114.89, 111.01, 110.12. EI-HRMS: Meas. m/z: 396.0256; Calc. Mass for $C_{23}H_{13}BrN_2$: 396.0257.

Synthesis of 3-bromo-2-(5,5-dioxido-10H-phenothiazin-10-yl)benzonitrile (BCPSO)

10H-phenothiazine (1.20 g, 6.02 mmol) was dissolved in 30 mL of anhydrous DMF. Sodium hydride (0.29 g, 7.23 mmol) dispersed in 60% mineral oil was added to the solution at 0 °C. After stirring for 30 minutes under N_2 atmosphere, 1-bromo-2-fluorobenzene (1.45 g, 7.23 mmol) was added. Then the reaction mixture was heated to refluxed and stirred for 12 hours. After cooling to room temperature, the reaction mixture was slowly poured into 300 mL of ice water while stirred. The mixture was then filtered, and the residue was dissolved in dichloromethane (60 mL), acetic acid (30 mL), and 30% hydrogen peroxide (H_2O_2, 2 mL). The mixture was stirred and reacted overnight at 60 °C. The reaction mixture was extracted with dichloromethane and further purified by Chromatography on a silica-gel column with dichloromethane-hexane (2/1, v/v) as eluent to afford the target product of BCPSO as white powders (1.28 g, 3.11 mmol) with 51.7% yield. ^{1}H NMR (400 MHz, $CDCl_3$) δ (TMS, ppm): 8.24 (dd, 2H), 8.18 (d, 1H), 7.98 (d, 1H), 7.66 (m, 1H), 7.51 – 7.45 (m, 2H), 7.35 (t, 2H), 6.40 (d, 2H). ^{13}C NMR (101 MHz, $CDCl_3$) δ 139.97, 139.92, 138.07, 134.20, 133.59, 131.81, 127.57, 124.44, 123.99, 123.50, 117.95, 115.55, 114.36. EI-HRMS: Meas. m/z: 409.9722; Calc. Mass for $C_{19}H_{11}BrN_2O_2S$: 409.9719.

Synthesis of 3,6-dibromo-9-hexyl-9H-carbazole (2BCzA)

2BCzA was synthesized according to the synthetic procedure of PC with 3,6-dibromo-9H-carbazole (1.00 g, 3.08 mmol), sodium hydride (0.37 g, 9.23 mmol) dispersed in 60% mineral oil, 1-bromohexane (0.61 g, 3.69 mmol), and 30 mL of anhydrous THF. The crude product was purified by Chromatography on a silica-gel column with hexane as eluent to afford the target product of 2BCzA as white powders (0.98 g, 2.40 mol) with 77.8% yield. ^{1}H NMR (400 MHz, DMSO-d_6) δ (ppm): 8.47 (s, 2H), 7.60 (s, 4H), 4.38 (t, 2H), 1.76 – 1.67 (m, 2H), 1.27 – 1.17 (m, 6H), 0.79 (t, 3H). ^{13}C NMR (101 MHz, DMSO-d_6) δ 139.06, 128.80, 123.43, 122.89, 111.67, 111.21, 42.50, 30.89, 28.34, 25.99, 21.97, 13.81. EI-HRMS: Meas. m/z: 406.9877; Calc. Mass for $C_{18}H_{19}Br_2N$: 406.9879.

Synthesis of 1-phenyl-1H-benzo[f]indole (*b*-CzA)

b-CzA was synthesized according to the synthetic procedure of PC with 5H-benzo[b]carbazole (50.0 mg, 230.1 mmol), sodium hydride (27.6 mg, 690.4 mmol) dispersed in 60% mineral oil, 1-bromohexane (45.6

mg, 276.2 mmol), and 10 mL of anhydrous THF. The crude product was purified by Chromatography on a silica-gel column with hexane as eluent to afford the target product of *b*-CzA as white powders (33.7 mg, 111.8 mmol) with 48.6% yield. ^{1}H NMR (400 MHz, DMSO-d_6) δ (ppm): 8.72 (s, 1H), 8.29 (d, *J* = 7.7 Hz, 1H), 8.05 (dd, *J* = 16.6, 8.3 Hz, 2H), 7.95 (s, 1H), 7.59 (d, *J* = 8.2 Hz, 1H), 7.56 – 7.46 (m, 2H), 7.42 – 7.36 (m, 1H), 7.23 (t, *J* = 7.3 Hz, 1H), 4.42 (t, *J* = 7.2 Hz, 2H), 1.83 (p, *J* = 7.2 Hz, 2H), 1.38 – 1.18 (m, 6H), 0.80 (t, *J* = 7.1 Hz, 3H). ^{13}C NMR (101 MHz, DMSO-d_6) δ 142.66, 139.87, 132.20, 128.24, 127.47, 127.38, 127.05, 125.01, 124.49, 122.49, 121.81, 121.04, 118.70, 118.51, 108.84, 103.54, 42.38, 31.02, 27.96, 26.21, 22.04, 13.86. EI-HRMS: Meas. m/z: 301.1828; Calc. Mass for $C_{22}H_{23}N$: 301.1825.

Supplementary Figures

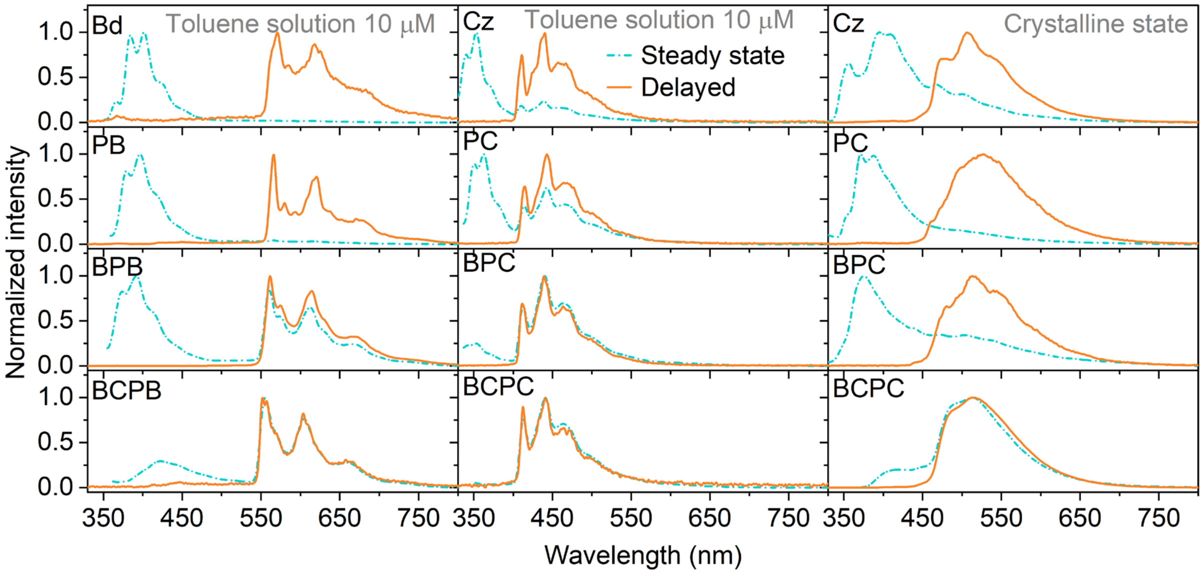

Supplementary Fig. 2 | Steady state and delayed spectra (delayed 10 ms) of the hosts (PC, BPC, and BCPC), guests (PB, BPB, and BCPB), carbazole (Cz), and benzoindole (Bd) in toluene solution and crystalline state at 77 K.

All these host molecules exhibited redshifted phosphorescence from dilute solution to the solid state. This phenomenon is attributed to the stabilization of triplet excitons by enhanced intermolecular interactions in the aggregated state, resulting in the formation of stabilized low-energy triplet excited states with redshifted aggregated phosphorescence[1].

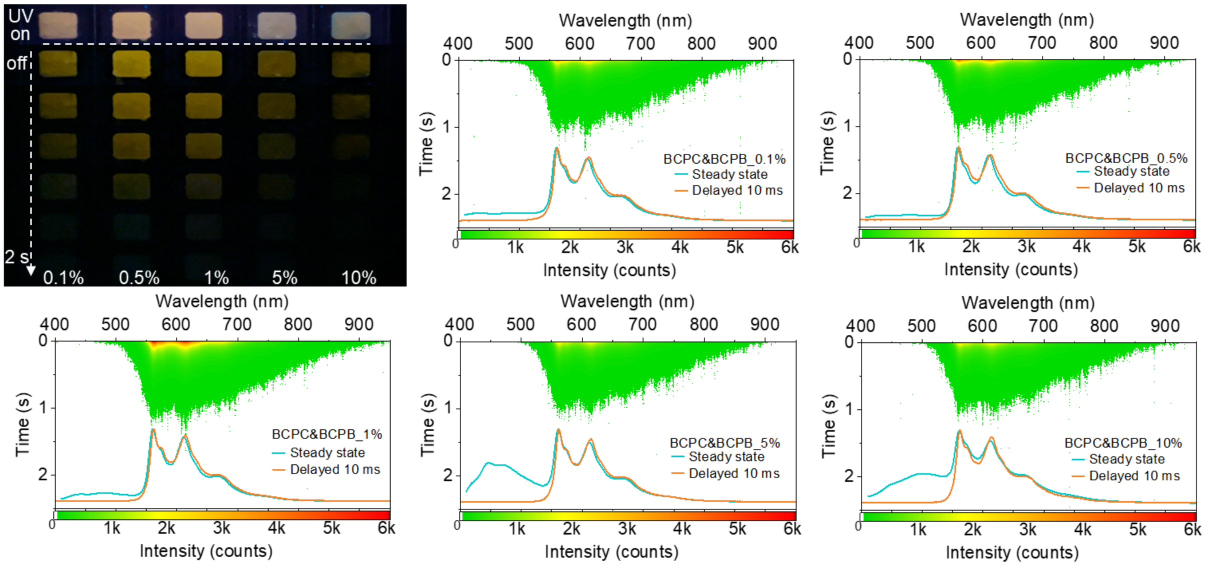

Supplementary Fig. 3 | Afterglow photographs and time-resolved phosphorescence spectra of BCPC&BCPB with varying doping ratios.

Among all the isostructural doping systems with different doping ratios, BCPC&BCPB_1% exhibited the best afterglow performance with the highest ratio of phosphorescence component (R_{Phos}) and phosphorescence quantum yields (Φ_{Phos}), and the longest phosphorescence lifetime (refer to Supplementary Fig. 3 and Supplementary Table 1). Hence, all the isostructural doping systems in this work adopt the doping ratio of 1% for experiments.

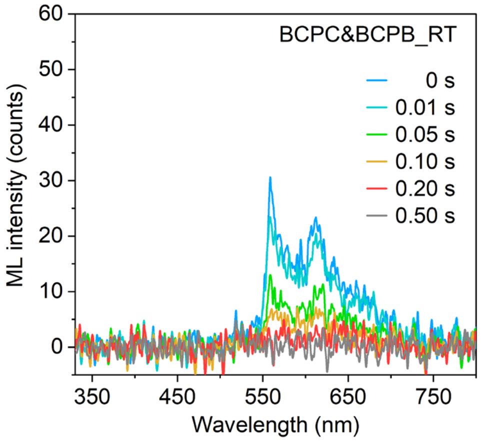

Supplementary Fig. 4 | Time-resolved emission spectra (TRES) of *p*ML for BCPC&BCPB at room temperature.

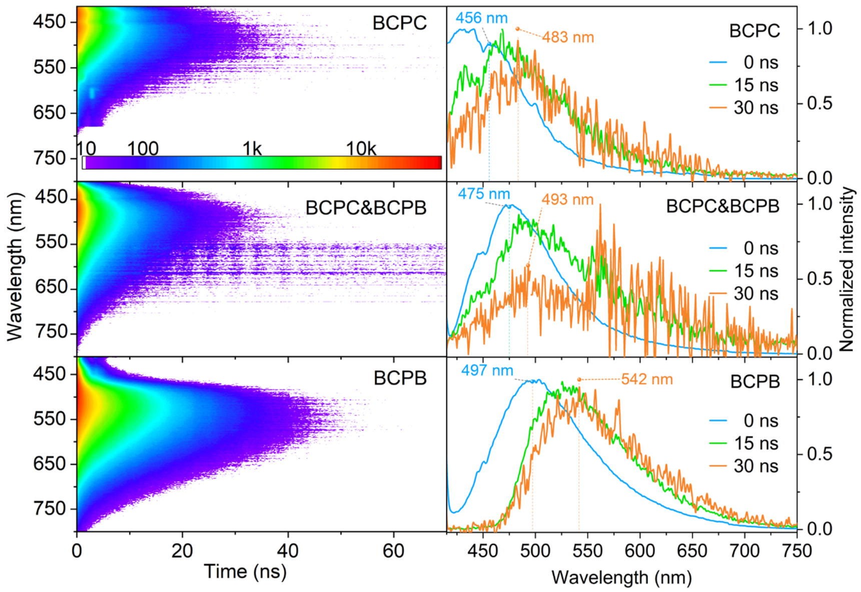

Supplementary Fig. 5 | TRES and the corresponding prompt spectra at different decay times of BCPC, BCPB, and BCPC&BCPB within 100 ns.

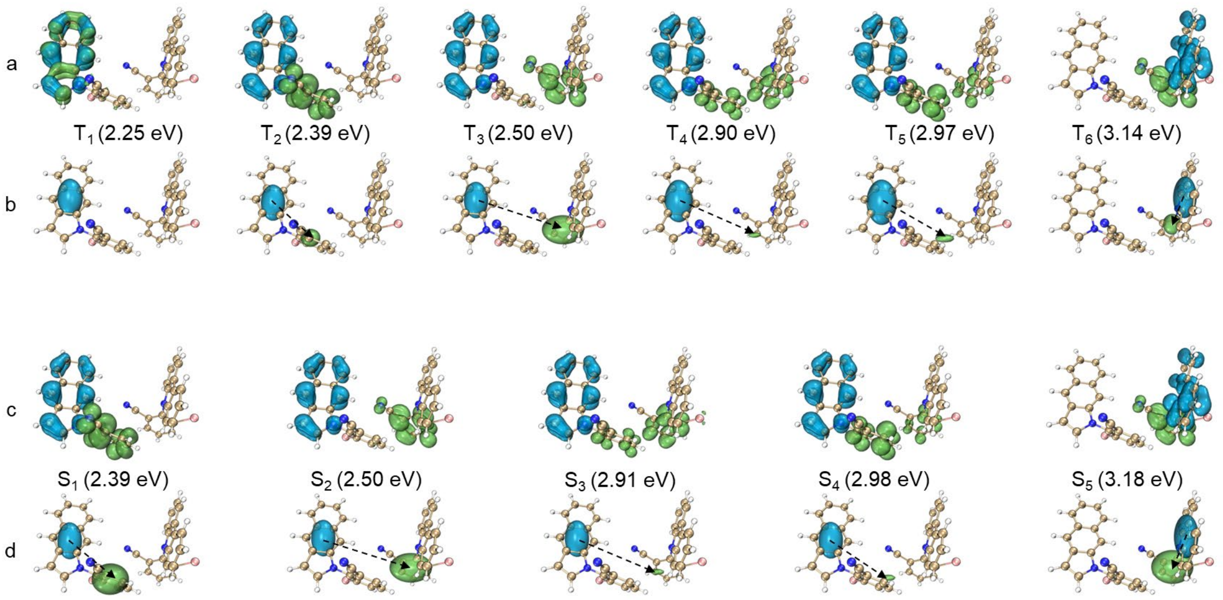

Supplementary Fig. 6 | TD-DFT calculation results of excited states of BCPC&BCPB optimized from BCPC single crystal: the isosurface maps of electron-hole density (**a** and **c**) and Cele-Chole difference (**b** and **d**). Blue and green isosurfaces correspond to hole and electron distribution, respectively. Chole and Cele are defined to describe the smooth distribution of holes (blue regions) and electrons (green regions). The dotted black arrows represent charge transfer between the hole and the electron center of mass.

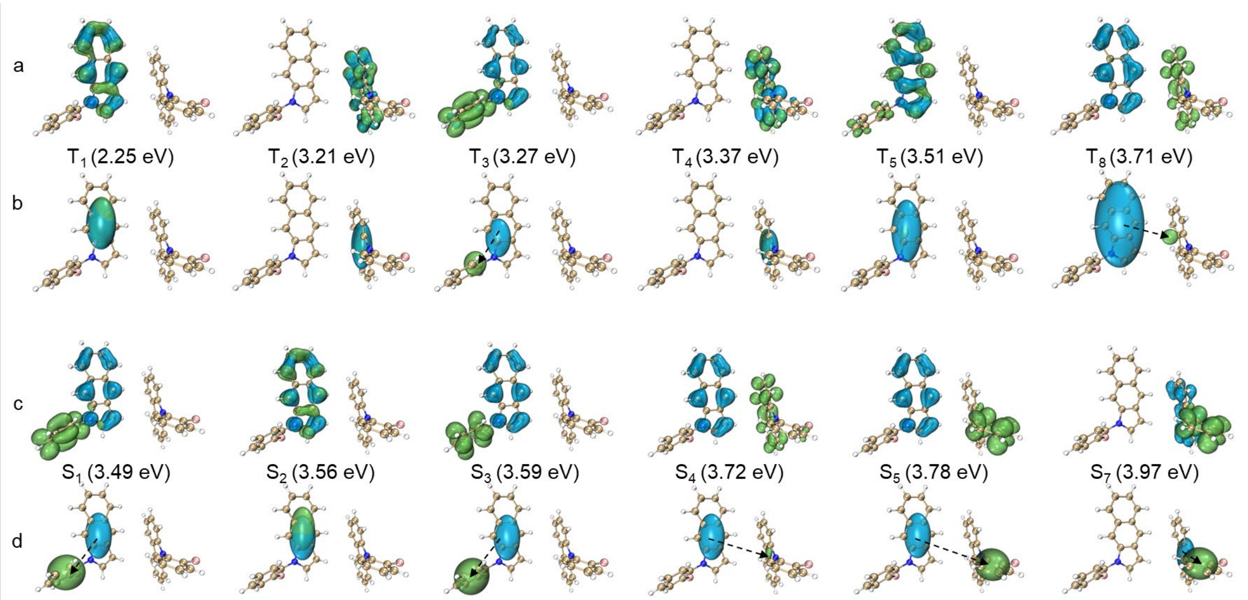

Supplementary Fig. 7 | TD-DFT calculation results of excited states of BPC&BPB optimized from BPC single crystal: the isosurface maps of electron-hole density (**a** and **c**) and Cele-Chole difference (**b** and **d**). Blue and green isosurfaces correspond to hole and electron distribution, respectively. Chole and Cele are defined to describe the smooth distribution of holes (blue regions) and electrons (green regions). The dotted black arrows represent charge transfer between the hole and the electron center of mass.

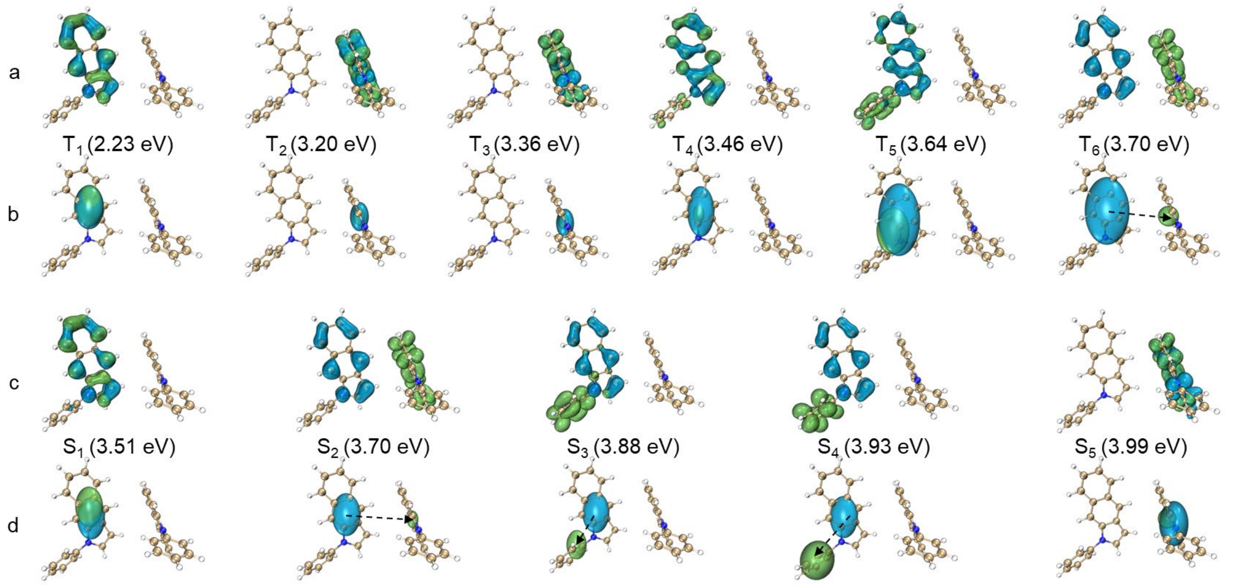

Supplementary Fig. 8 | TD-DFT calculation results of excited states of PC&PB optimized from PC single crystal: the isosurface maps of electron-hole density (**a** and **c**) and Cele-Chole difference (**b** and **d**). Blue and green isosurfaces correspond to hole and electron distribution, respectively. Chole and Cele are defined to describe the smooth distribution of holes (blue regions) and electrons (green regions). The dotted black arrows represent charge transfer between the hole and the electron center of mass.

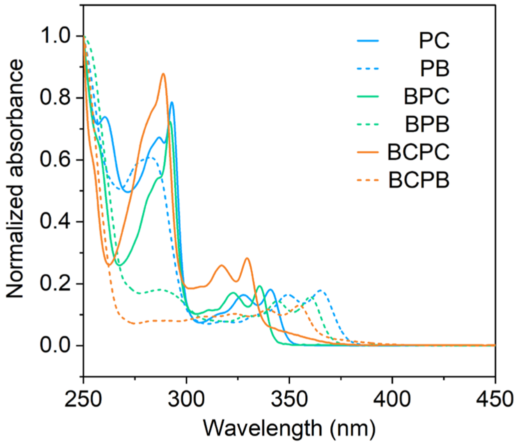

Supplementary Fig. 9 | UV-vis absorption spectra of the host and guest molecules in DCM solution (50 μM).

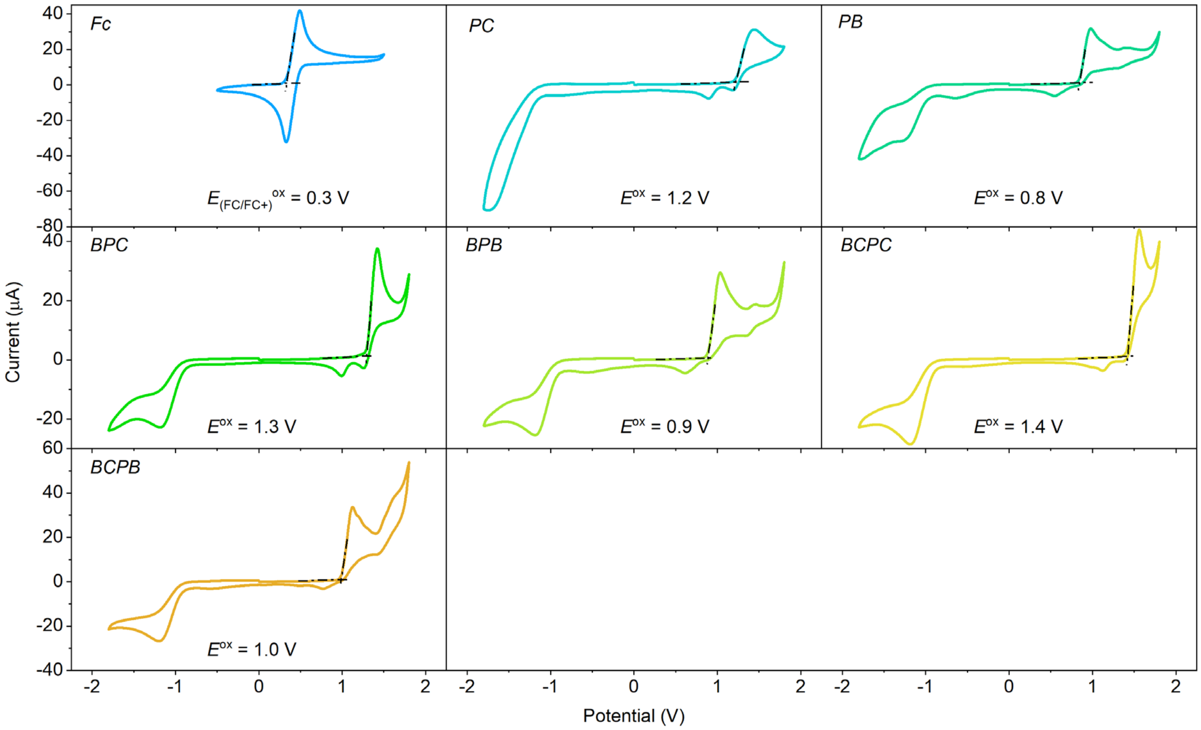

Supplementary Fig. 10 | Cyclic voltammetry curves of the host and guest molecules in DCM solution (10 μM).

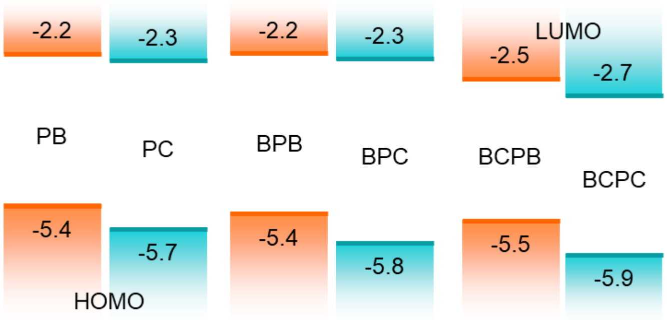

Supplementary Fig. 11 | HOMO and LUMO of the hosts (PC, BPC, and BCPC) and their isostructural guests (PB, BPB, and BCPB).

The values of the highest occupied molecular orbital (HOMO) and lowest unoccupied molecular orbital (LUMO) energy levels for these host and guest molecules can be calculated from the absorption and cyclic voltammetry data, as per the formulas (1)-(3). Here, E^{ox} represents the oxidation onset potential, E_g is the energy gap of the HOMO and LUMO within a molecule, and λ_{abs} is the initial absorption wavelength from the long-wavelength region. The small differences (ΔE < 0.3 eV) in lowest unoccupied molecular orbital (LUMO) energy levels between the host and guest of these doping systems could potentially lead to the reversion of charge separation[2,3]. This indirectly suggested that charge diffusion is limited in these isostructural doping systems.

$$\boldsymbol{E_{HOMO} = -[E^{ox} - E^{ox}_{(FC/FC^+)} + 4.8]} \quad (1)$$

$$\boldsymbol{E_g = \frac{hc}{\lambda_{abs}} = \frac{1240}{\lambda_{abs}}} \quad (2)$$

$$\boldsymbol{E_{LUMO} = E_{HOMO} - E_g} \quad (3)$$

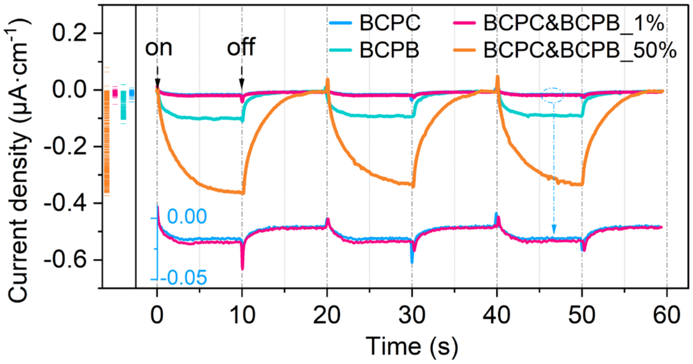

Supplementary Fig. 12 | Photocurrent density variation with (on) and without (off) irradiation of a white-light source for BCPC, BCPB, and BCPC&BCPB.

A clear enhancement in photocurrent density was observed in BCPC&BCPB_50%, in contrast to the marginal increase seen in BCPC&BCPB_1% compared with BCPC alone. This, combined with the non-power-law emission decay of ultralong lifetimes (refer to Fig. 2b) and small LUMO energy level differences ($\Delta E < 0.3$ eV) in between the host and guest (see Supplementary Fig. 11), suggested that while charge separation within the host-guest pairs can indeed occur, the efficiency of charge diffusion was notably limited in these isostructural doping systems at a low doping ratio. Consequently, the generation of intermediate charge-separated states and subsequent gradual charge recombination are scarcely realized in these isostructural doping systems.

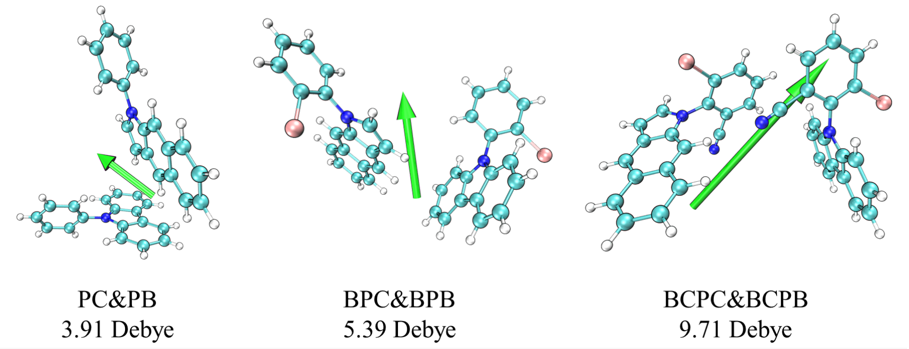

Supplementary Fig. 13 | Dipole moments of the host/guest pairs of PC&PB, BPC&BPB, and BCPC&BCPB.

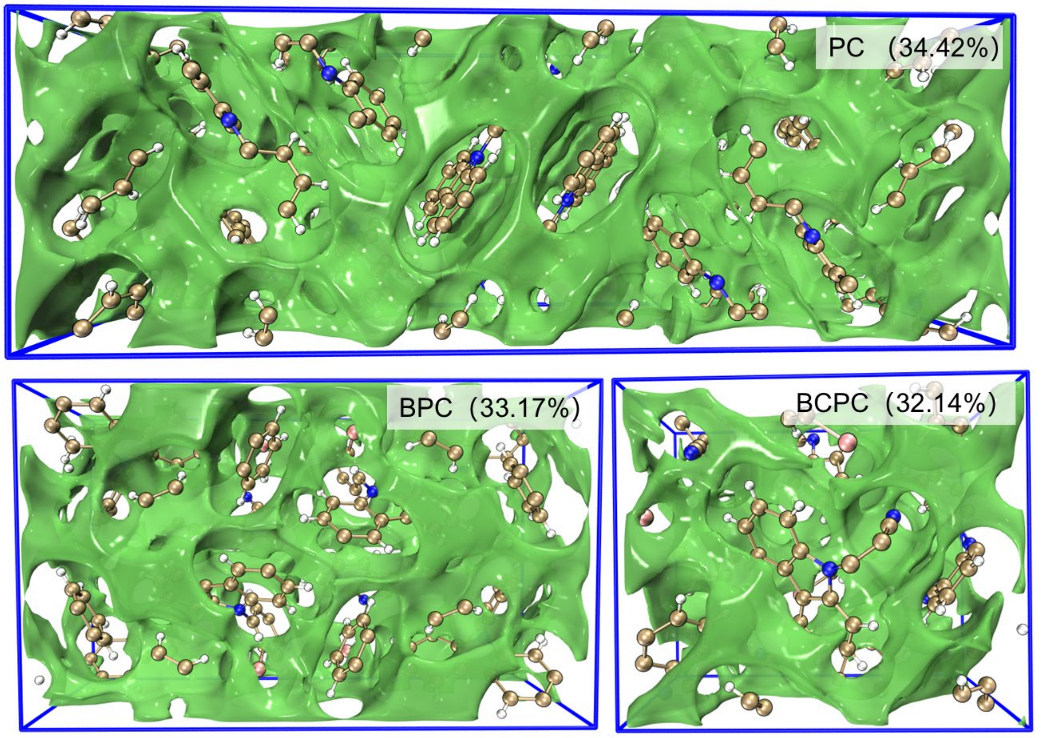

Supplementary Fig. 14 | Crystal voids analysis for unit cells of PC, BPC, and BCPC single crystals.

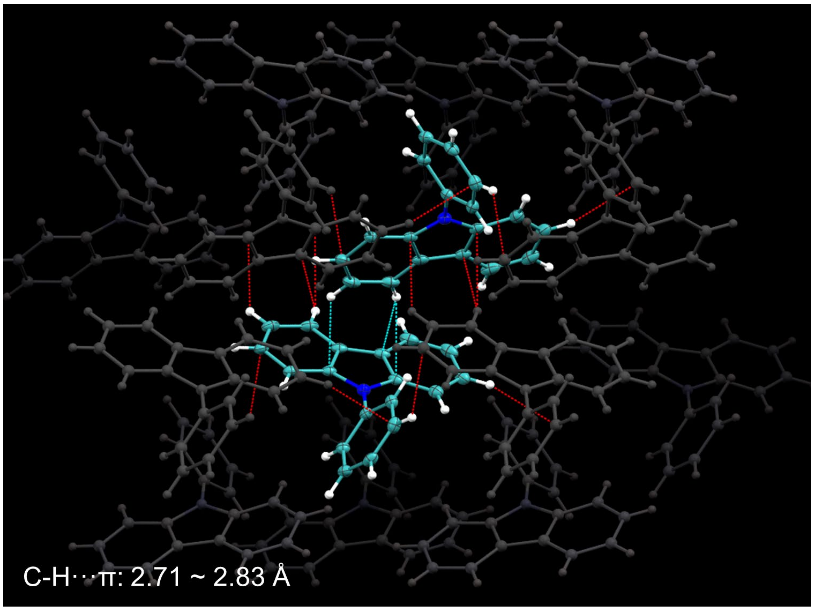

Supplementary Fig. 15 | Intermolecular interactions outside the PC dimer and packing structure of the single crystal.

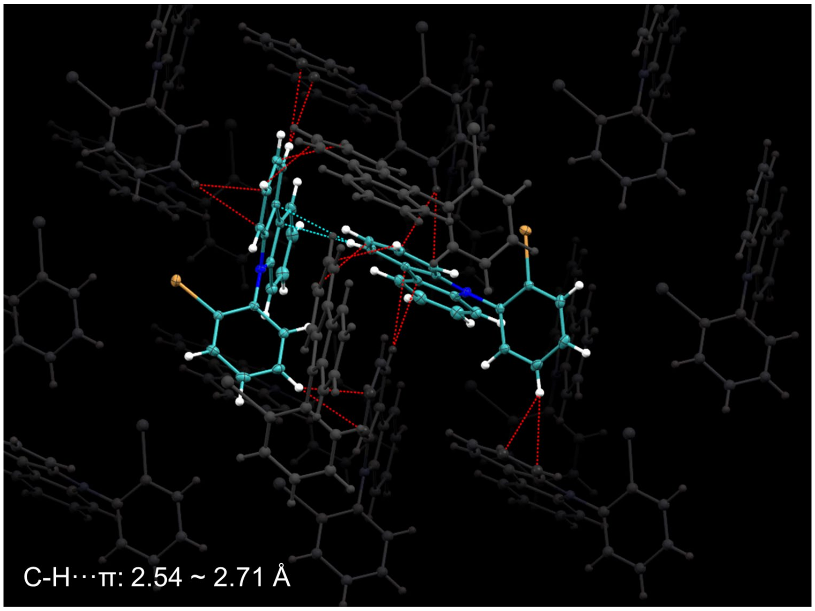

Supplementary Fig. 16 | Intermolecular interactions outside the BPC dimer and packing structure of the single crystal.

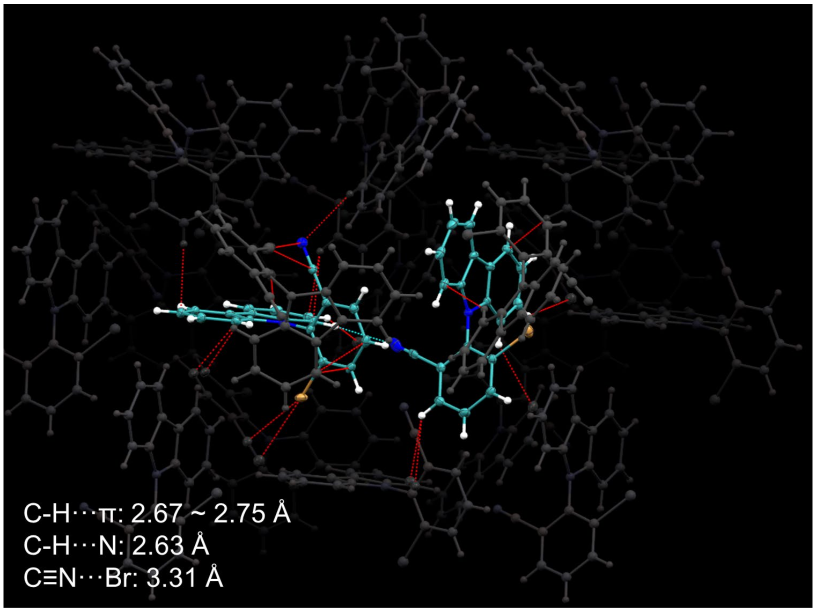

Supplementary Fig. 17 | Intermolecular interactions outside the BCPC dimer and packing structure of the single crystal.

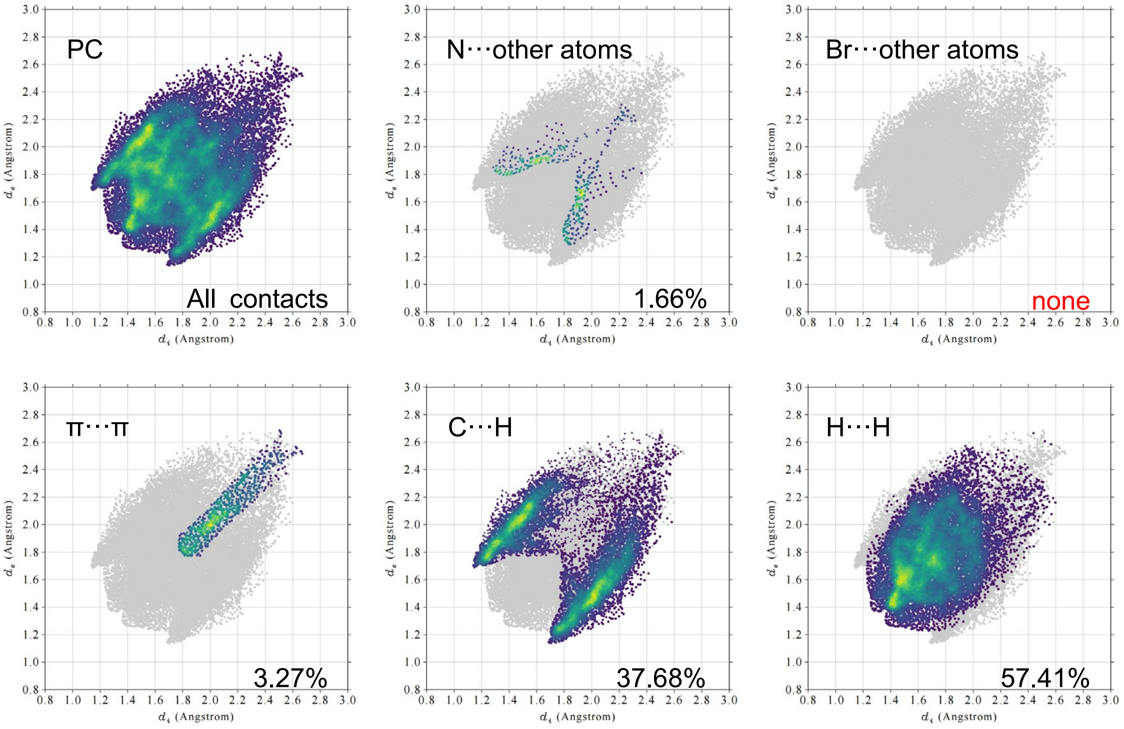

Supplementary Fig. 18 | Fingerprint plot analyses based on PC single crystal.

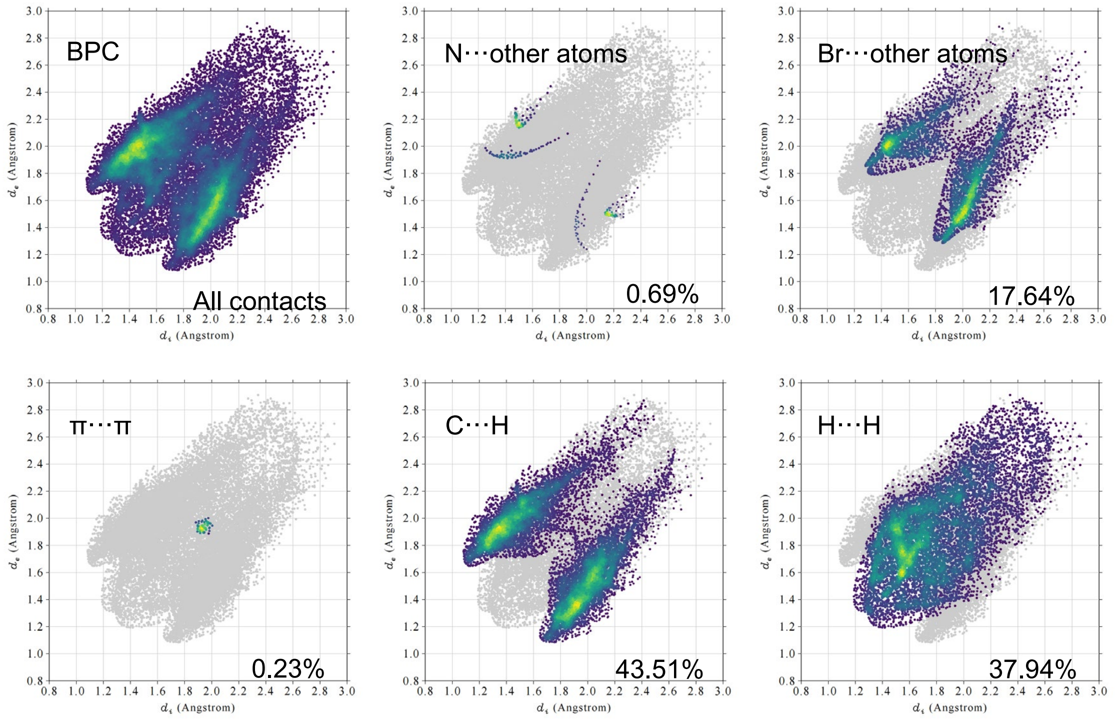

Supplementary Fig. 19 | Fingerprint plot analyses based on BPC single crystal.

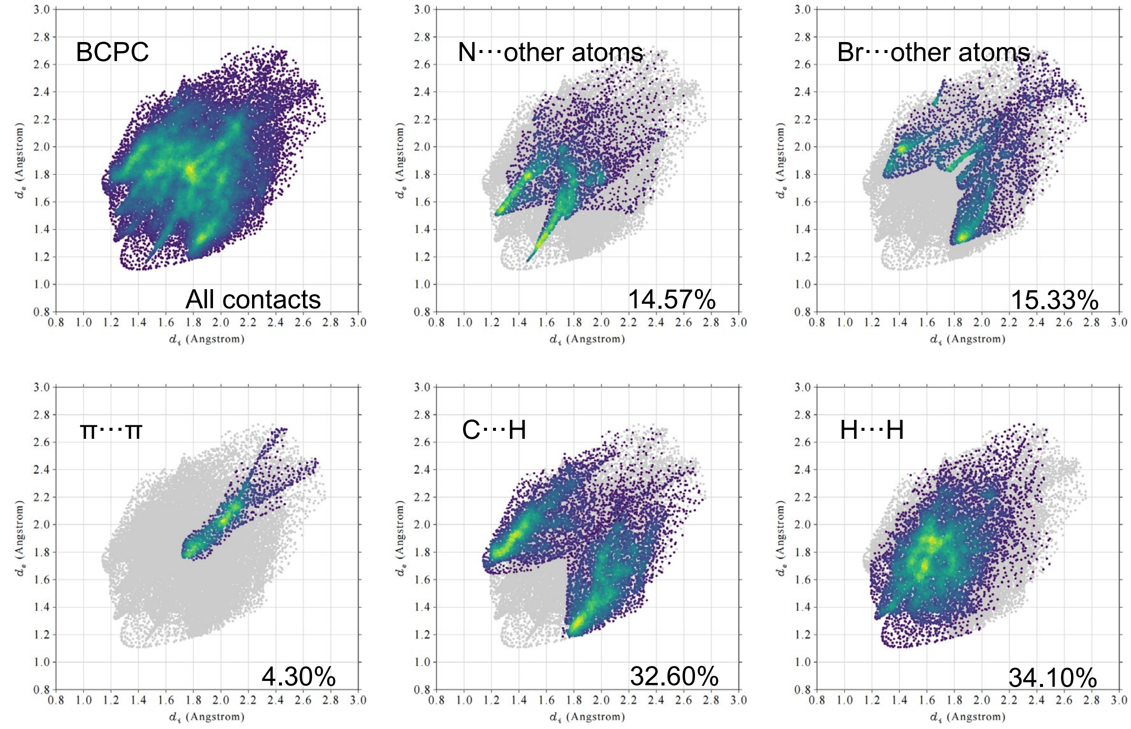

Supplementary Fig. 20 | Fingerprint plot analyses based on BCPC single crystal.

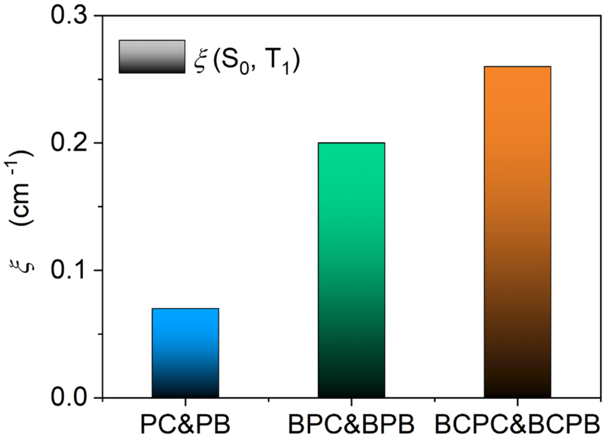

Supplementary Fig. 21 | Spin-orbital coupling matrix elements (ξ) between T_1 and S_0 of PC&PB, BPC&BPB, and BCPC&BCPB.

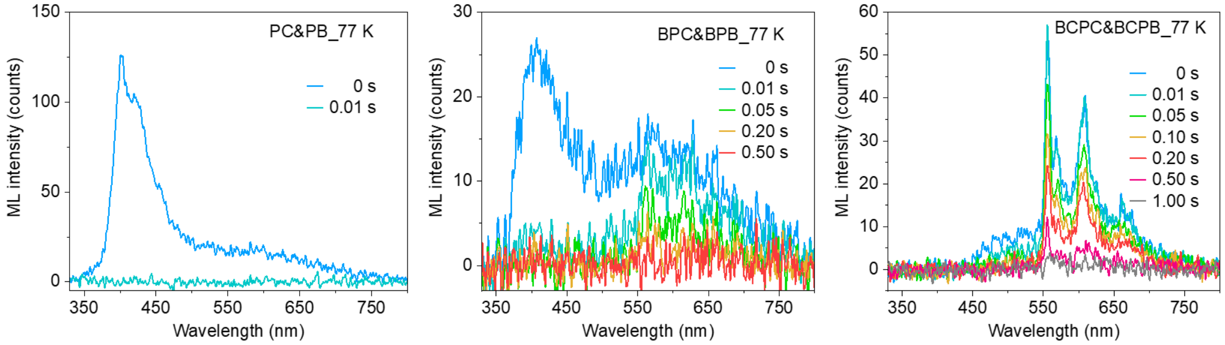

Supplementary Fig. 22 | TRES of ML for PC&PB, BPC&BPB, and BCPC&BCPB at 77 K.

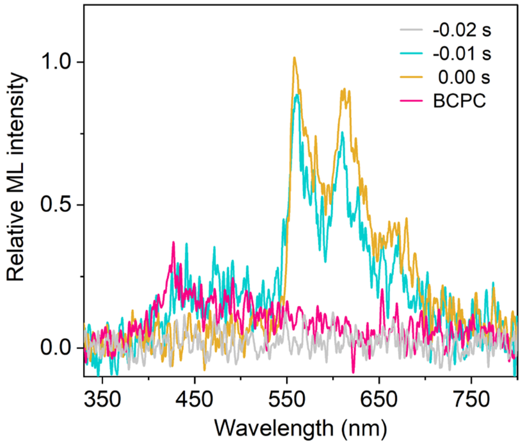

Supplementary Fig. 23 | ML spectra of BCPC&BCPB at different collection times.

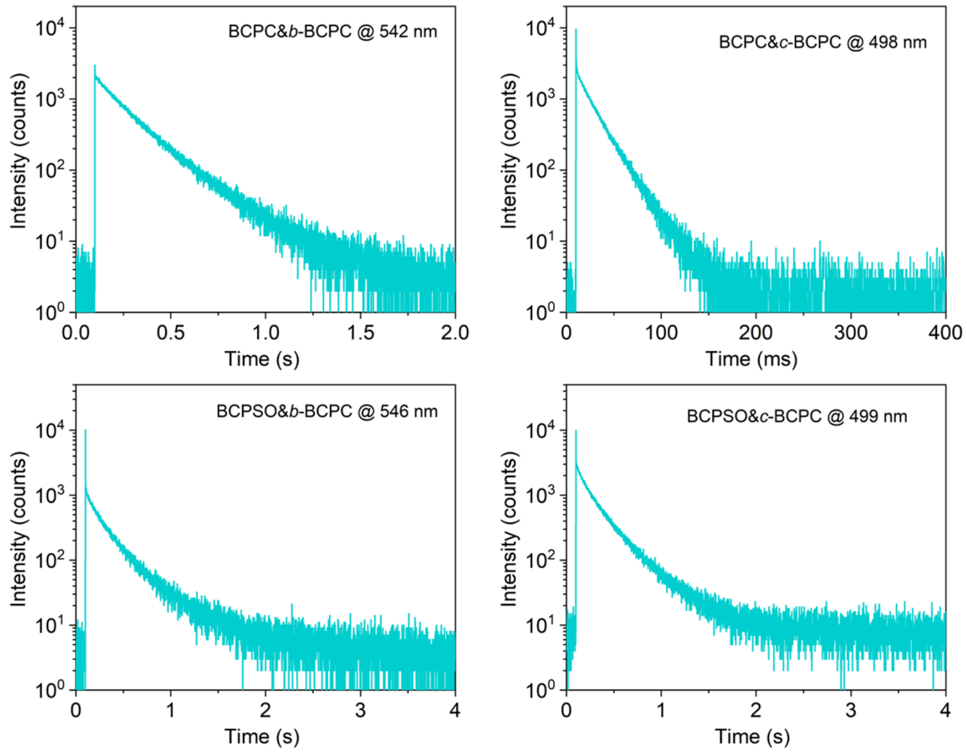

Supplementary Fig. 24 | PL decay profiles of the phosphorescence for BCPC&*b*-BCPC, BCPC&*c*-BCPC, BCPSO&*b*-BCPC, and BCPSO&*c*-BCPC (λ_{ex} = 365 nm).

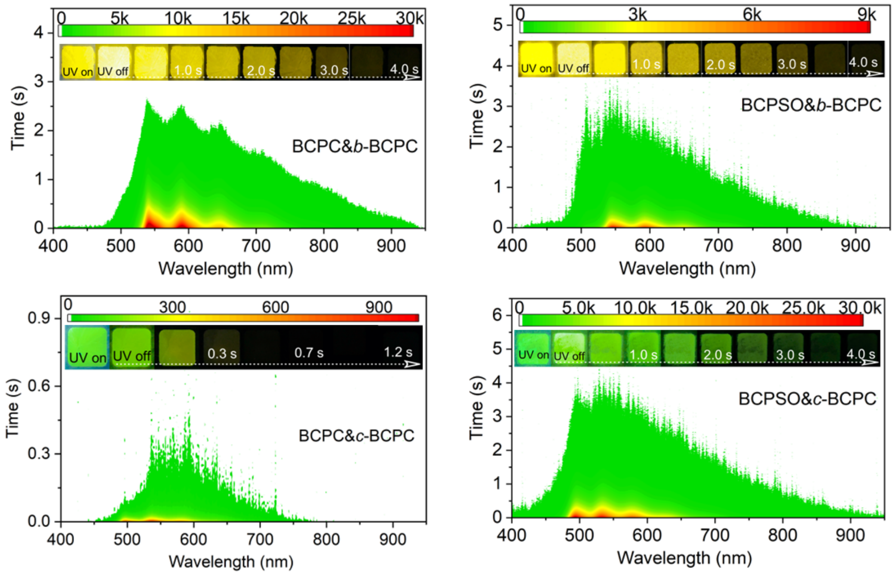

Supplementary Fig. 25 | TRES mapping of BCPC&*b*-BCPC, BCPC&*c*-BCPC, BCPSO&*b*-BCPC, and BCPSO&*c*-BCPC. Inset: Luminescent photographs of the isostructural doping systems under UV on and UV off.

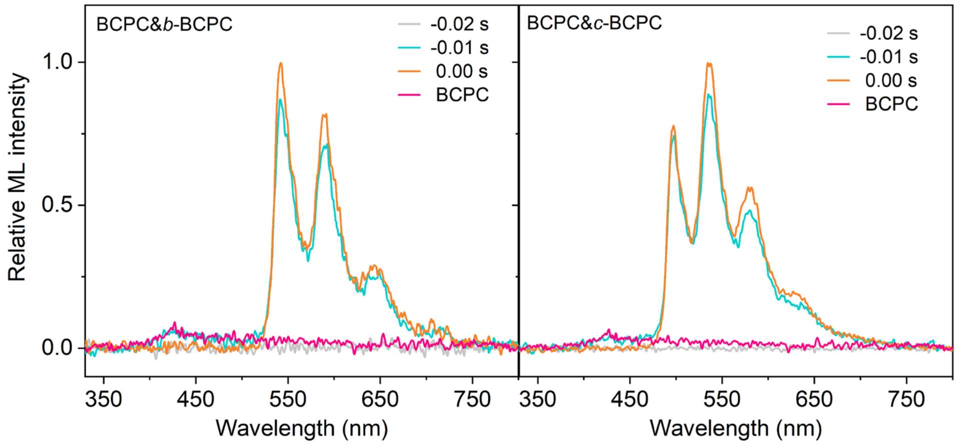

Supplementary Fig. 26 | ML spectra of BCPC&*b*-BCPC and BCPC&*c*-BCPC at different collection times.

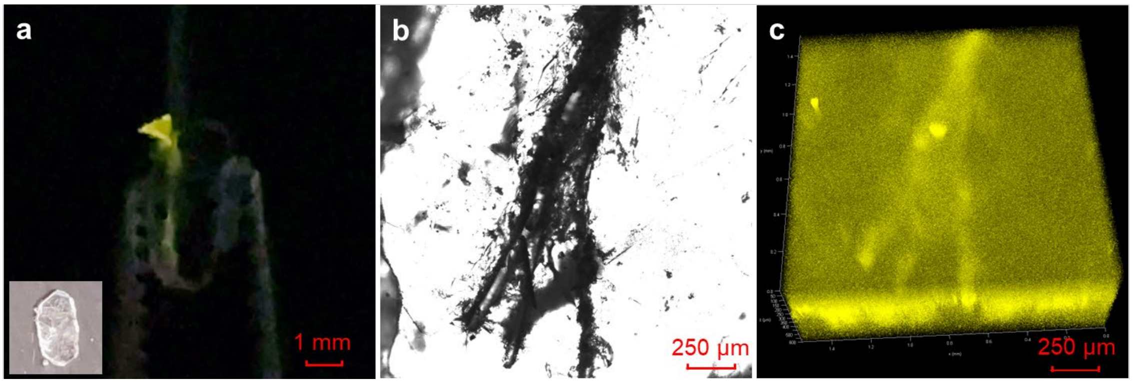

Supplementary Fig. 27 | *p*ML photograph (a), confocal microscope image (b), and 3D confocal fluorescence image (c) of the fracture surface for BCPC&*b*-BCPC single crystal. Please take note that the uniform yellow fluorescent background was observed due to the optical excitation of the molecules under confocal microscope.

Intense *p*ML is observed exclusively on the scratched marks when a sharp knife scratches the crystal surface. 3D confocal fluorescence imaging measurements were recorded to assess the distribution of mechanical stimulation applied to the crystal. The entire crystal emitted uniform yellow fluorescence when excited by a 405 nm laser, with a more pronounced luminescence signal observed at the fracture surfaces (see Supplementary Fig. 27c). It reveals that, apart from the fractured surface, the other parts of the single crystal are uniform. Combined with the *p*ML photograph and the confocal fluorescence images of the fractured surface of the BCPC&*b*-BCPC single crystal, it can be concluded that mechanical stimulation only acts on the fracture surface, generating *p*ML in situ.

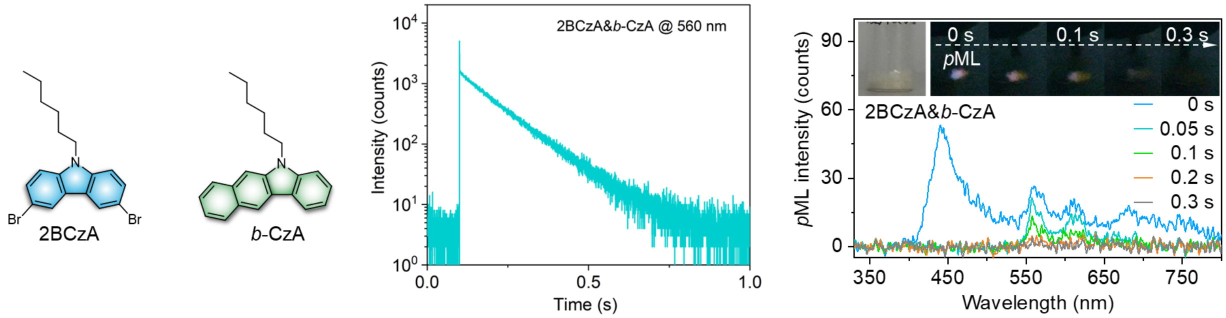

Supplementary Fig. 28 | Chemical structures of 2BCzA and *b*-CzA, PL decay profile and TRES of *p*ML for 2BCzA&*b*-CzA. Inset: *p*ML images of the doped material.

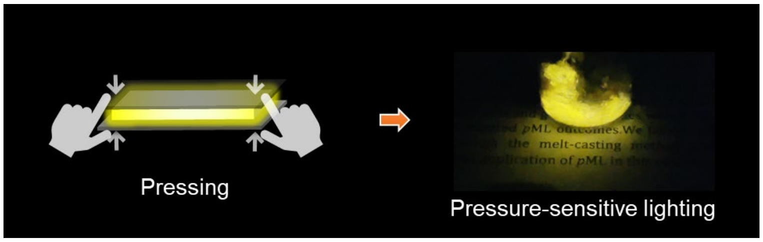

Supplementary Fig. 29 | Demonstration of application of BCPC&*b*-BCPC for pressure-sensitive lighting.

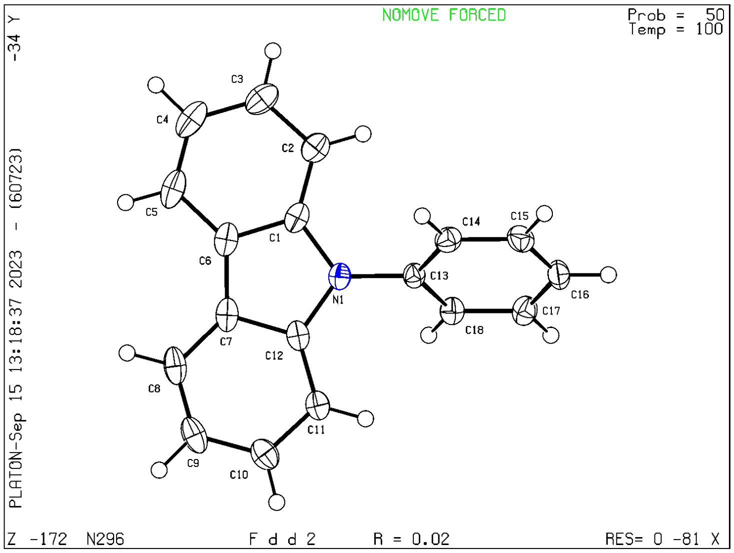

Supplementary Fig. 30 | Single crystal structure of PC.

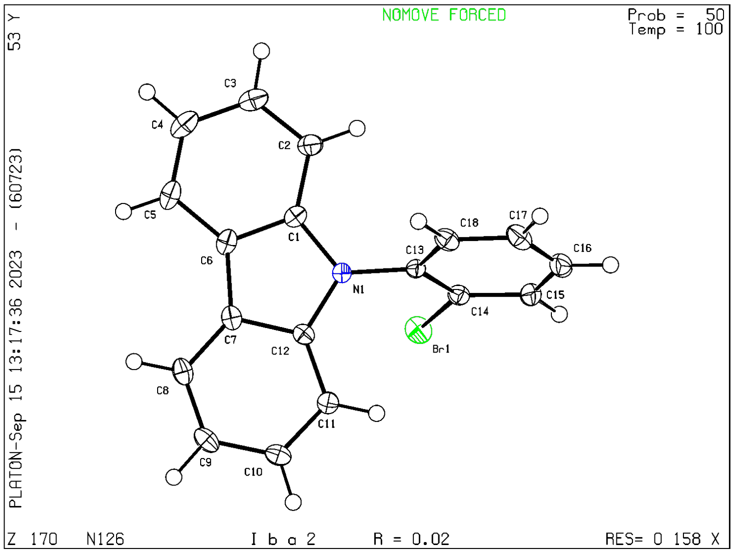

Supplementary Fig. 31 | Single crystal structure of BPC.

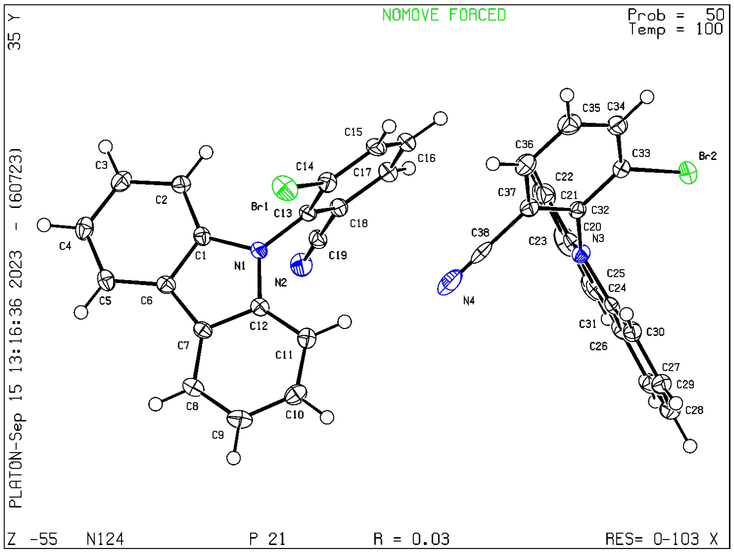

Supplementary Fig. 32 | Single crystal structure of BCPC.

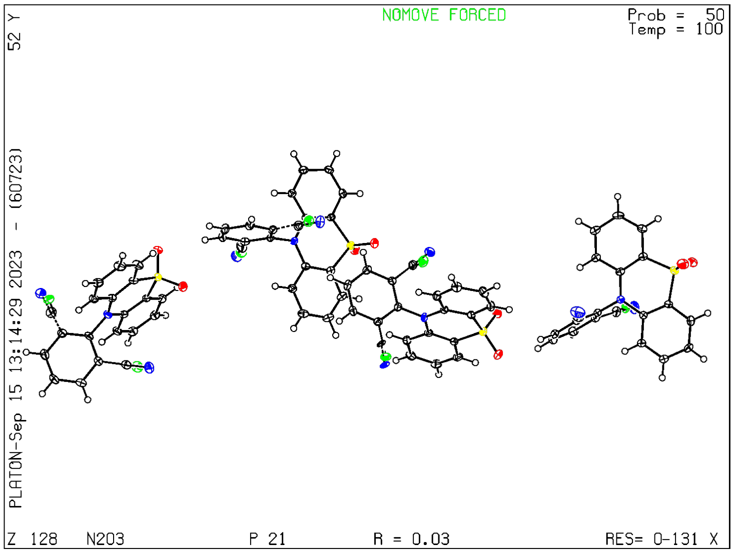

Supplementary Fig. 33 | Single crystal structure of BCPSO.

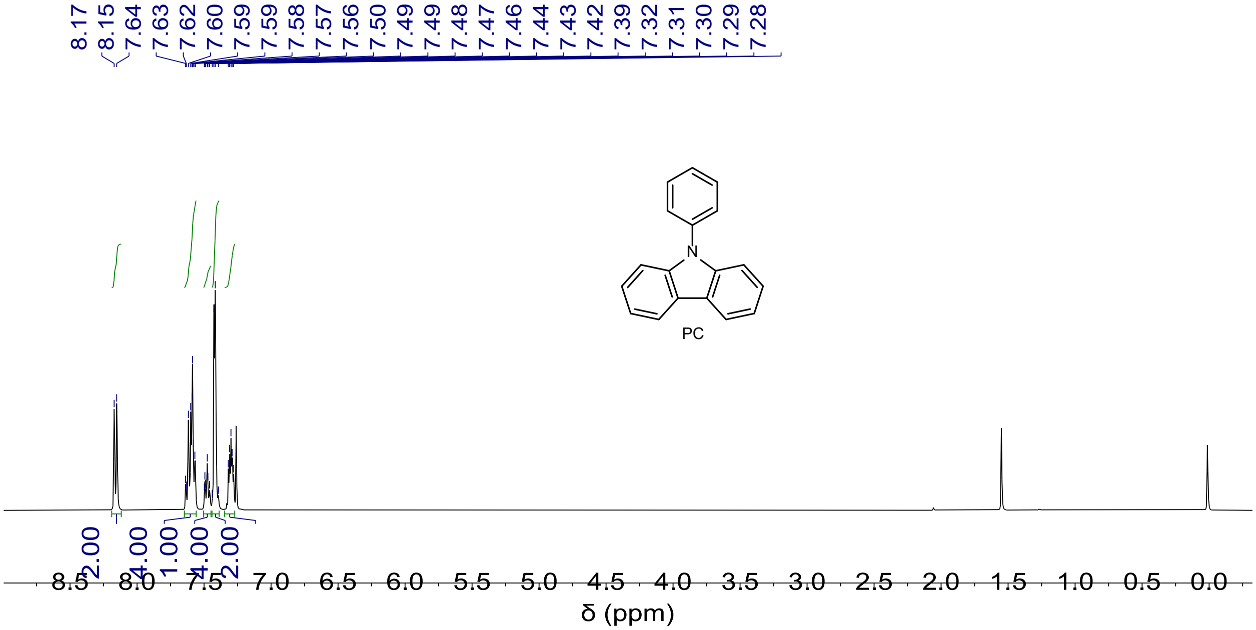

Supplementary Fig. 34 | ^{1}H NMR spectrum of PC (400 Mz, $CDCl_3$).

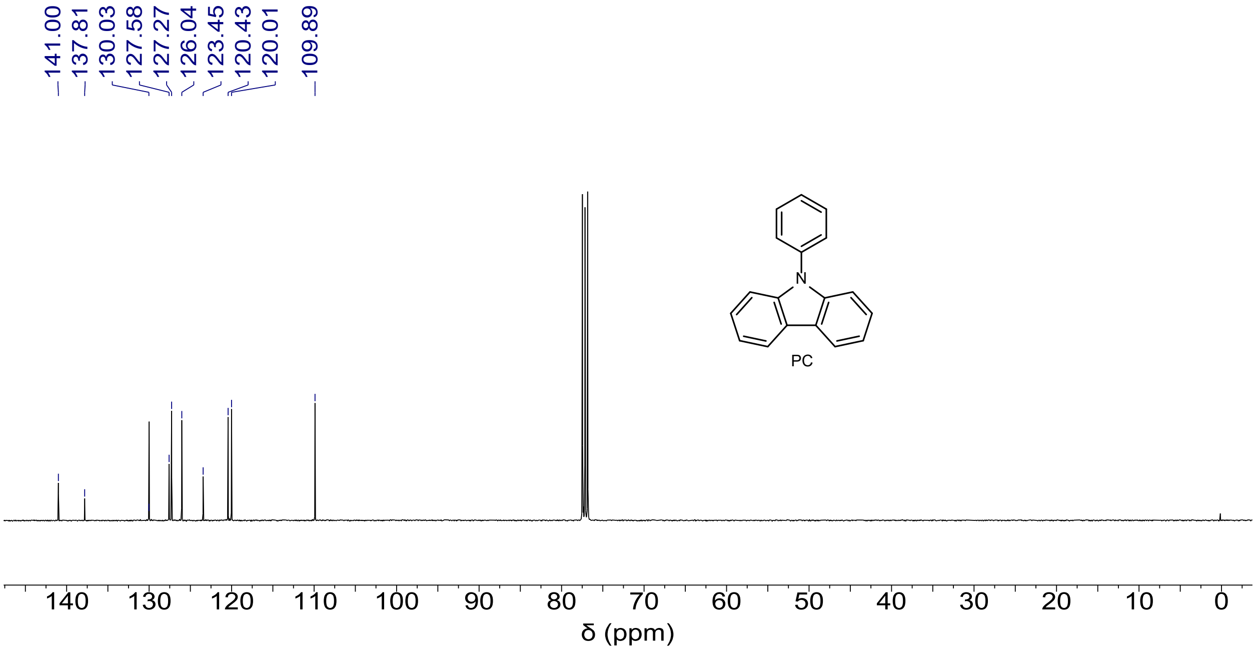

Supplementary Fig. 35 | ^{13}C NMR spectrum of PC (101 Mz, $CDCl_3$).

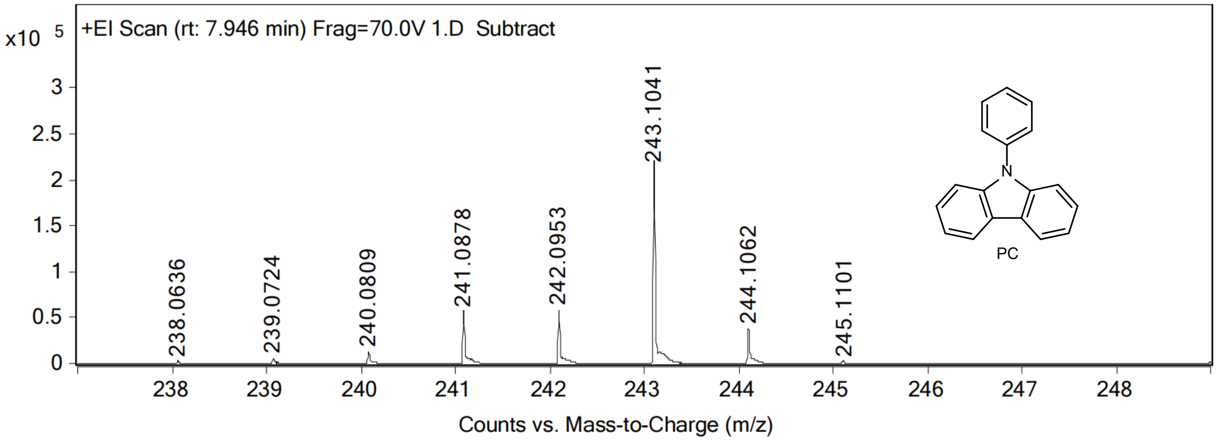

Supplementary Fig. 36 | EI-HRMS of PC.

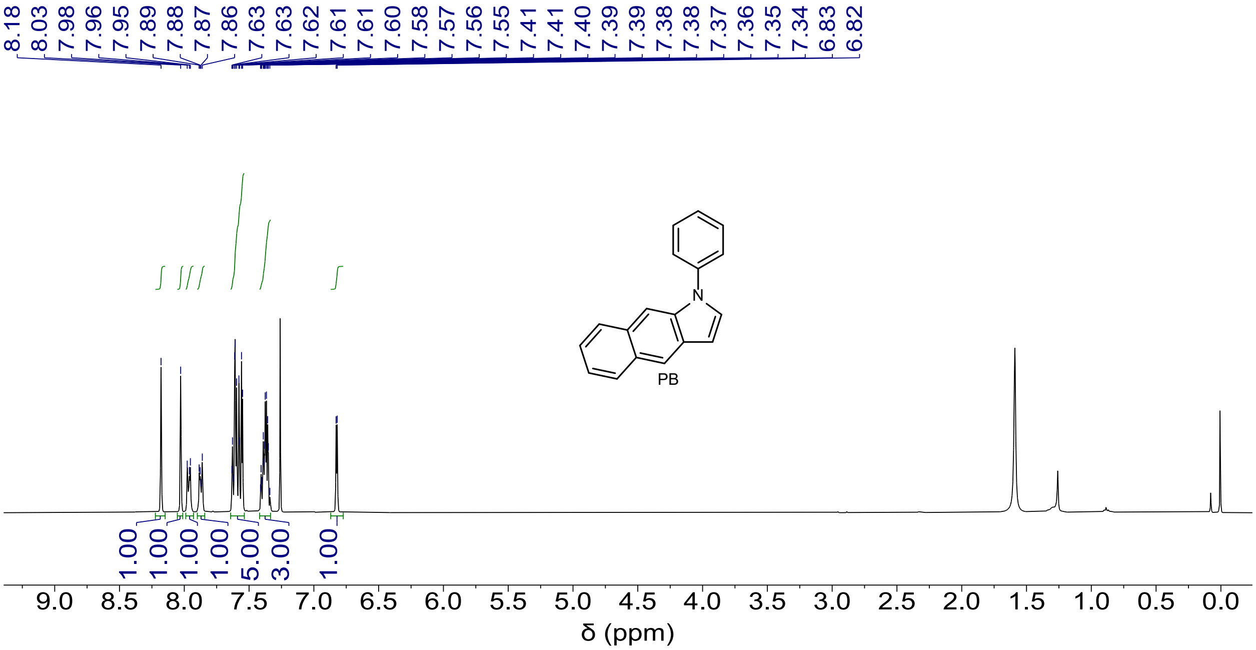

Supplementary Fig. 37 | ^{1}H NMR spectrum of PB (400 Mz, $CDCl_3$).

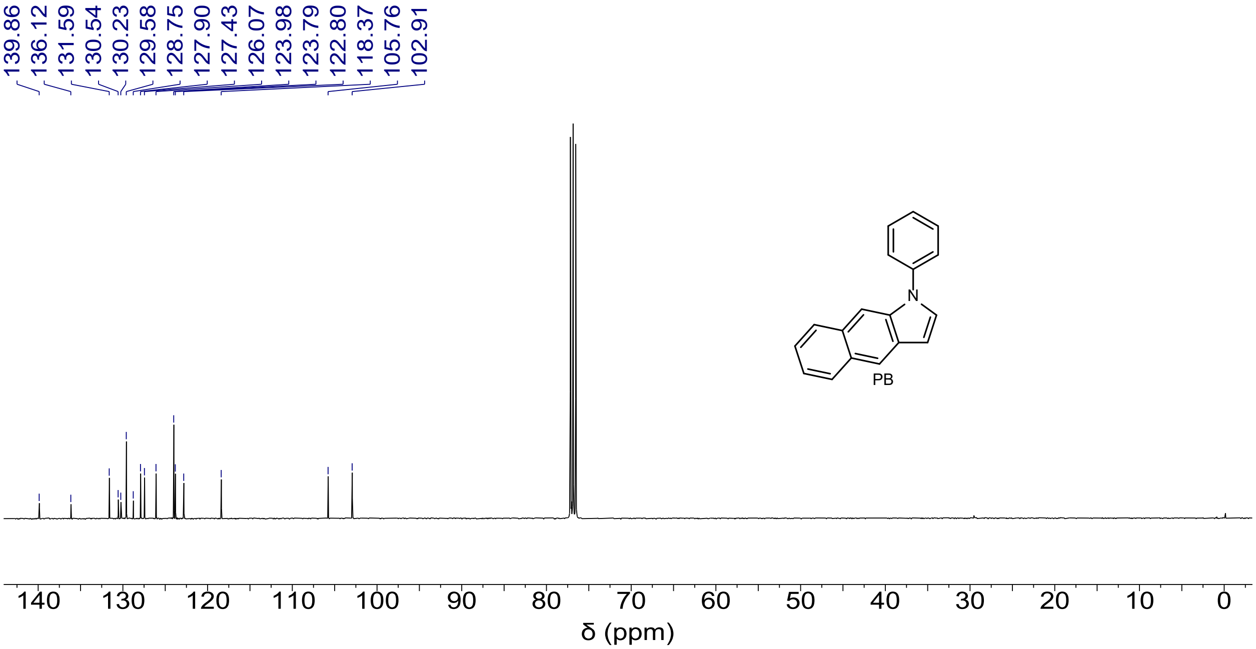

Supplementary Fig. 38 | ^{13}C NMR spectrum of PB (101 Mz, $CDCl_3$).

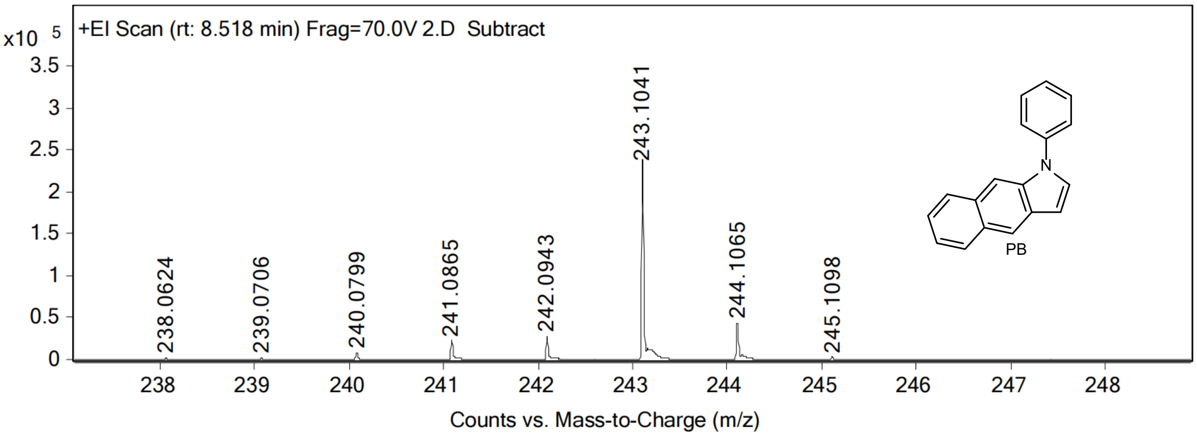

Supplementary Fig. 39 | EI-HRMS of PB.

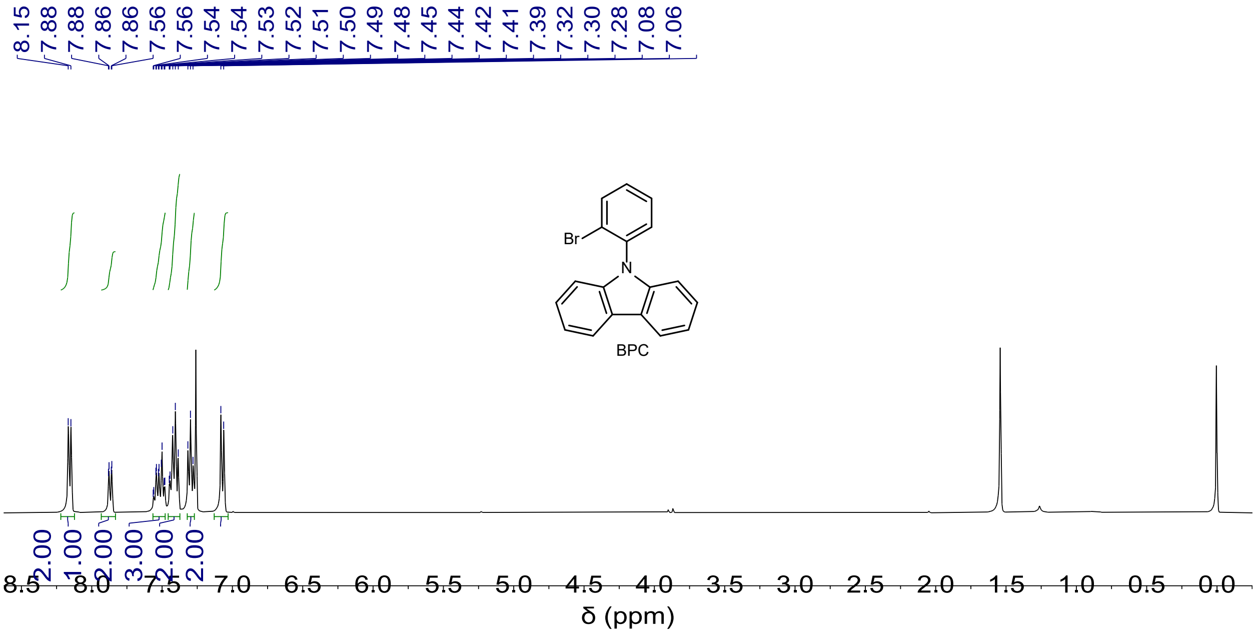

Supplementary Fig. 40 | ^{1}H NMR spectrum of BPC (400 Mz, $CDCl_3$).

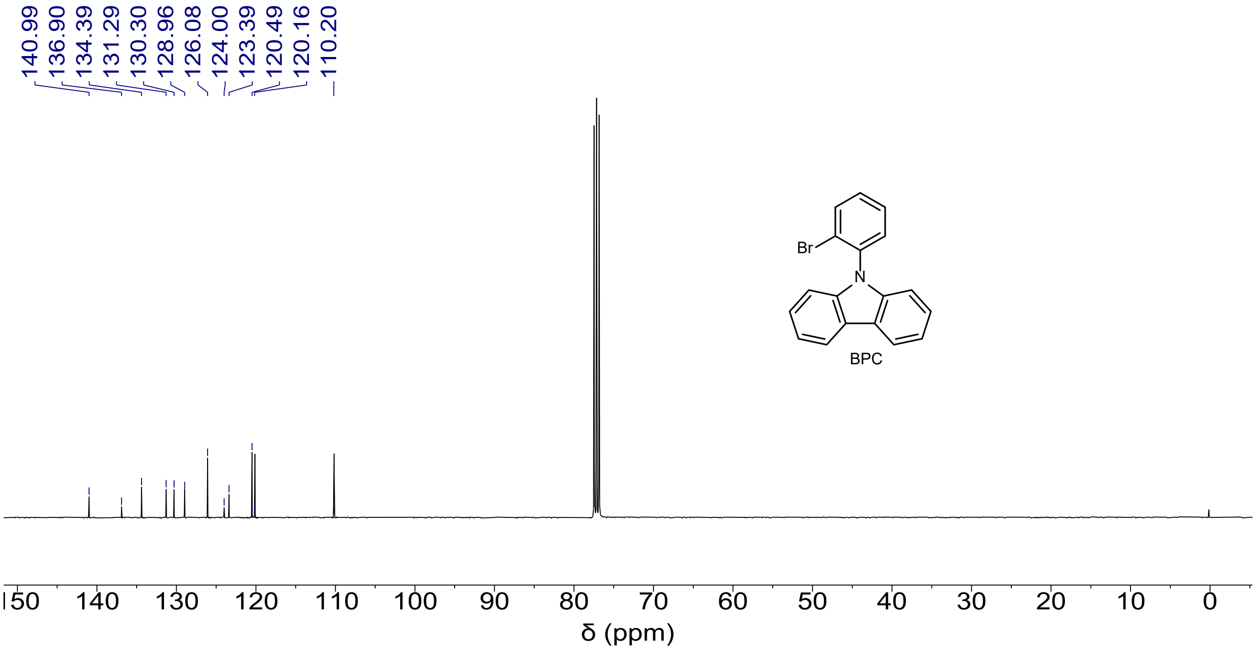

Supplementary Fig. 41 | ^{13}C NMR spectrum of BPC (101 Mz, $CDCl_3$).

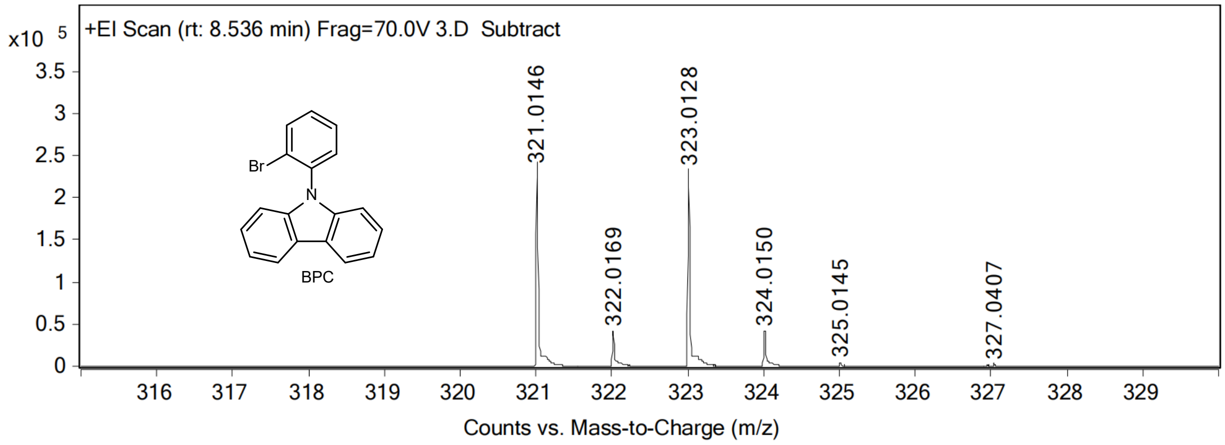

Supplementary Fig. 42 | EI-HRMS of BPC.

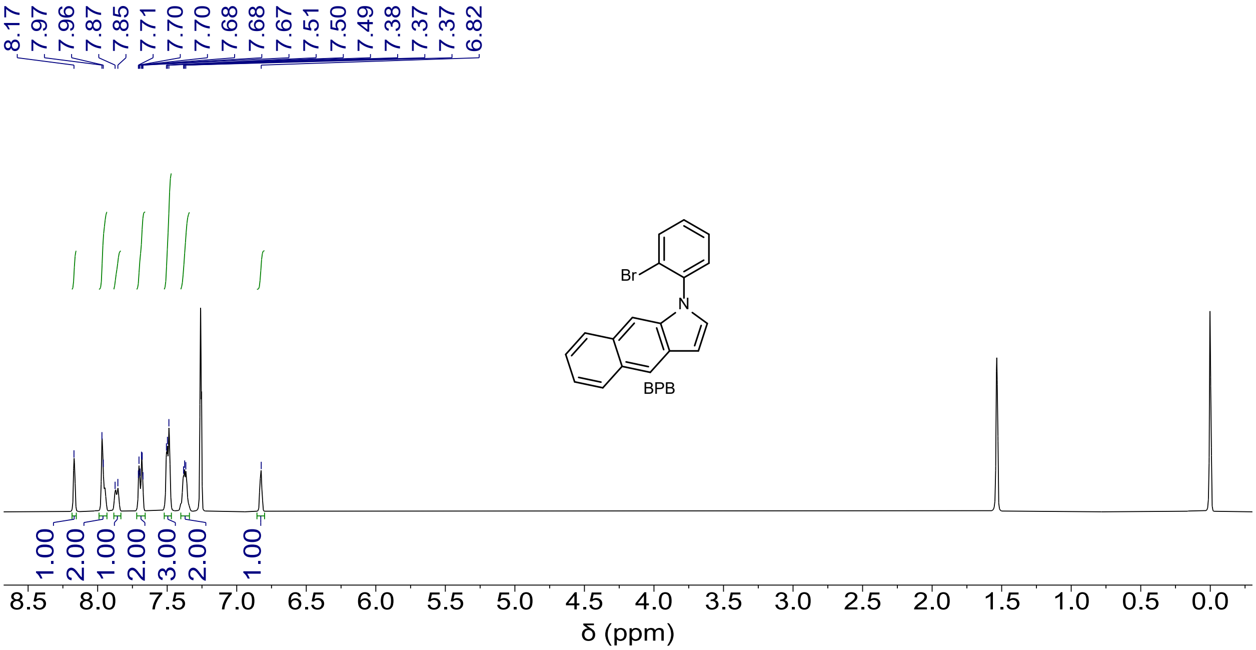

Supplementary Fig. 43 | ^{1}H NMR spectrum of BPB (400 Mz, $CDCl_3$).

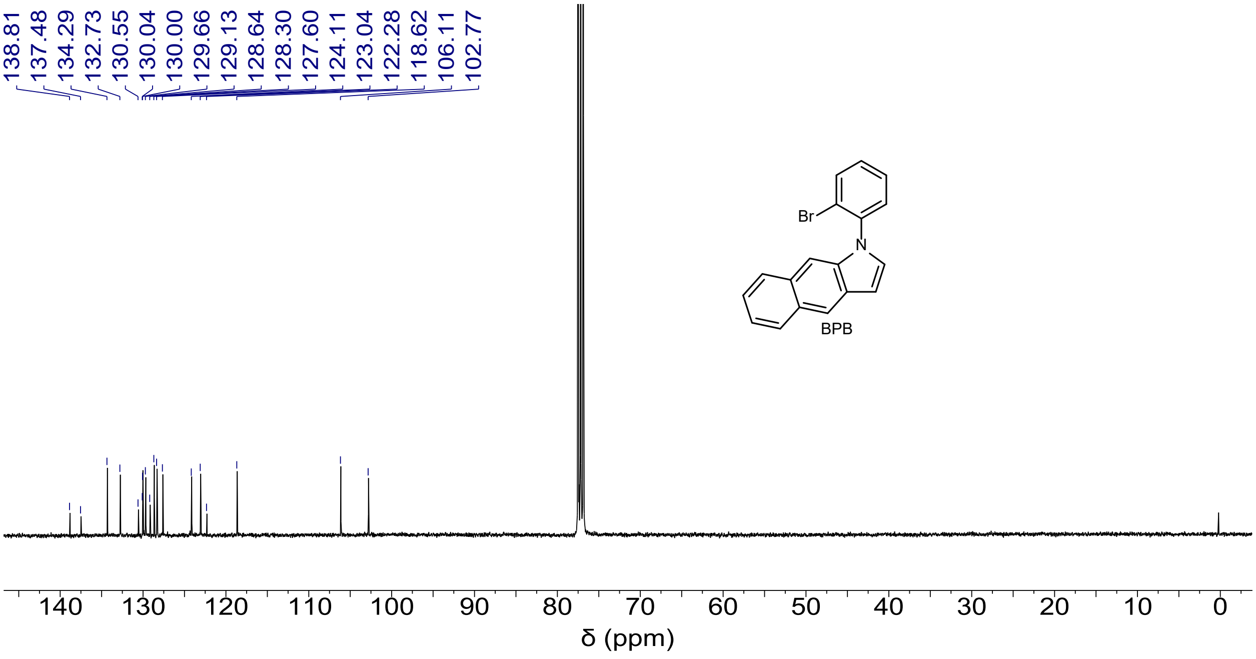

Supplementary Fig. 44 | ^{13}C NMR spectrum of BPB (101 Mz, $CDCl_3$).

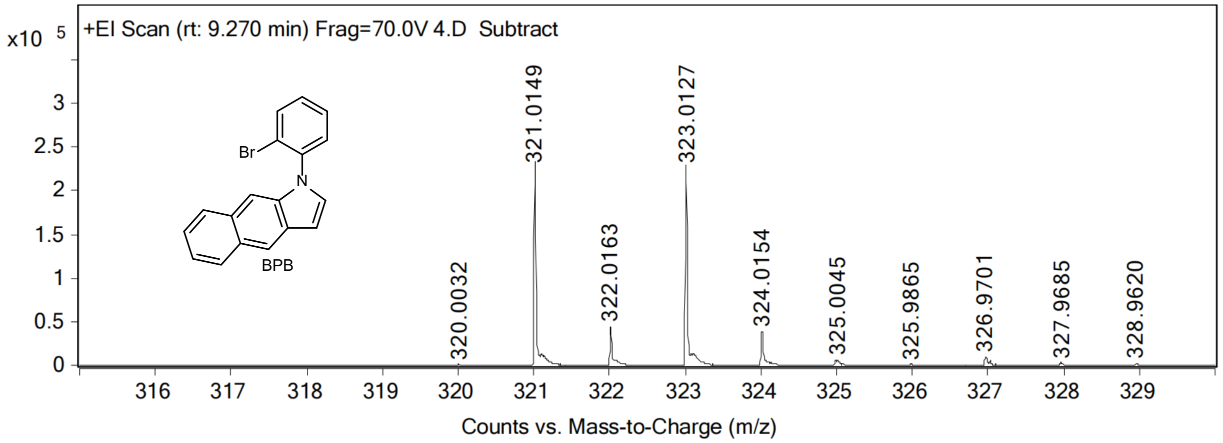

Supplementary Fig. 45 | EI-HRMS of BPB.

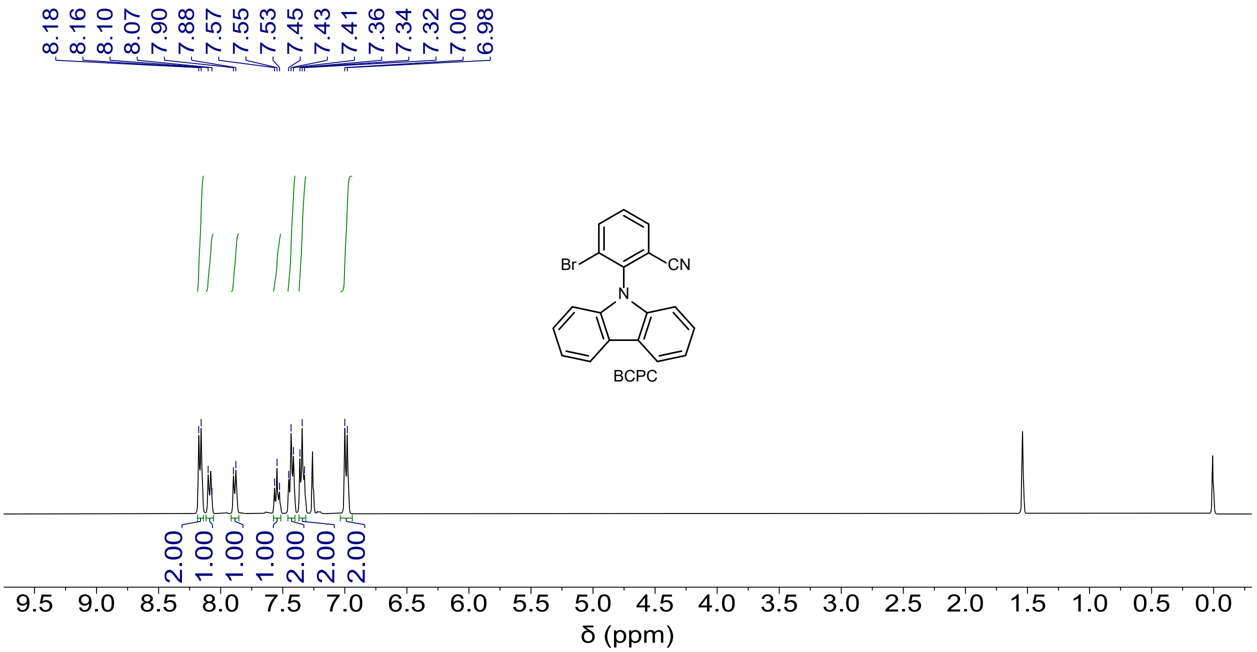

Supplementary Fig. 46 | ^{1}H NMR spectrum of BCPC (400 Mz, $CDCl_3$).

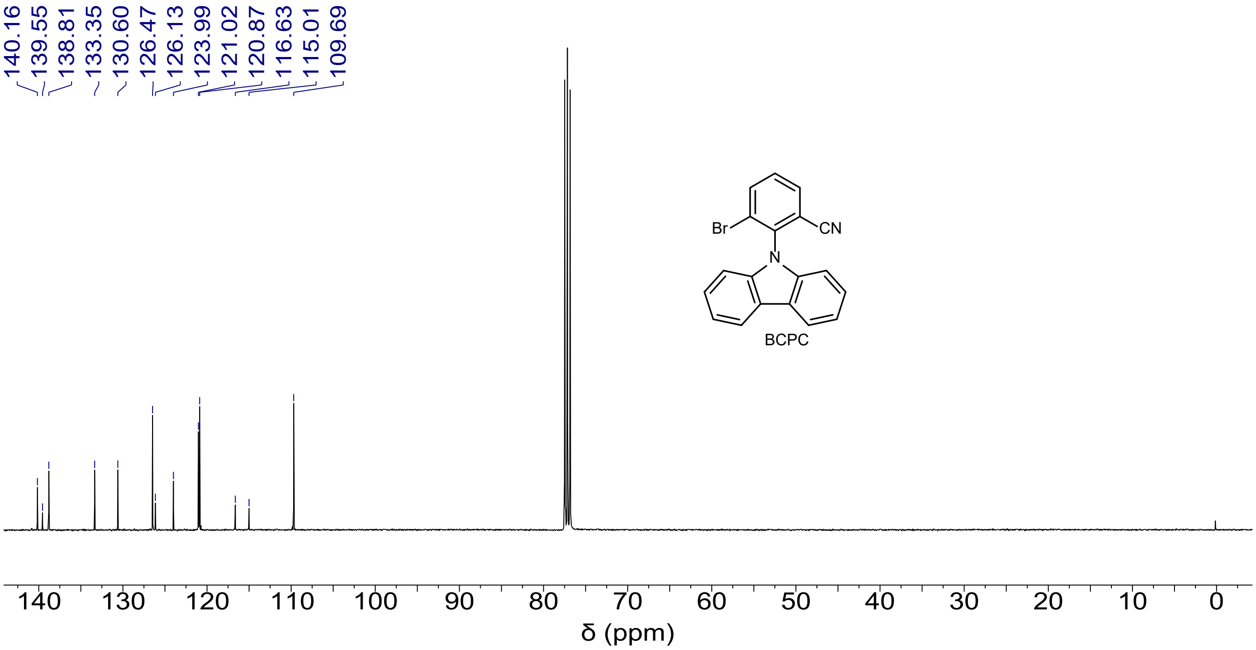

Supplementary Fig. 47 | ^{13}C NMR spectrum of BCPC (101 Mz, $CDCl_3$).

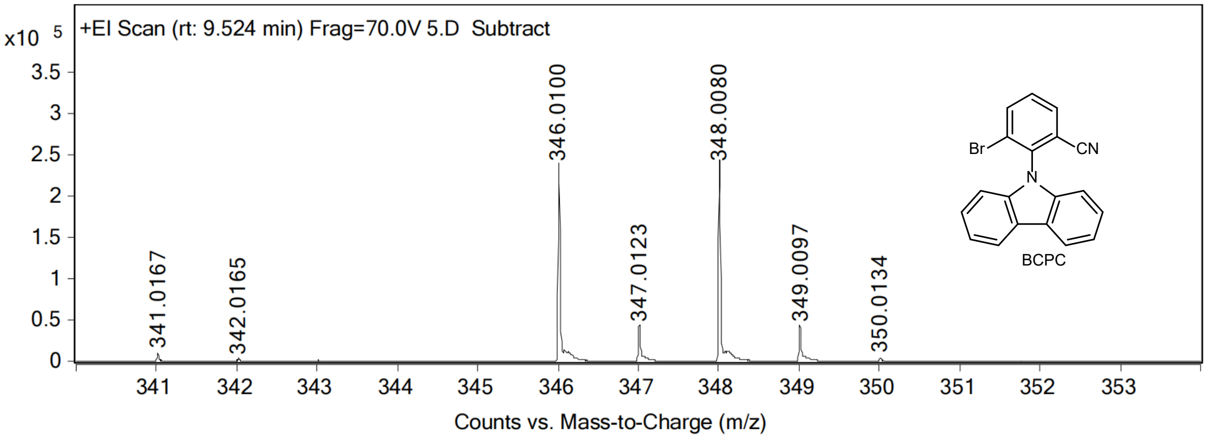

Supplementary Fig. 48 | EI-HRMS of BCPC.

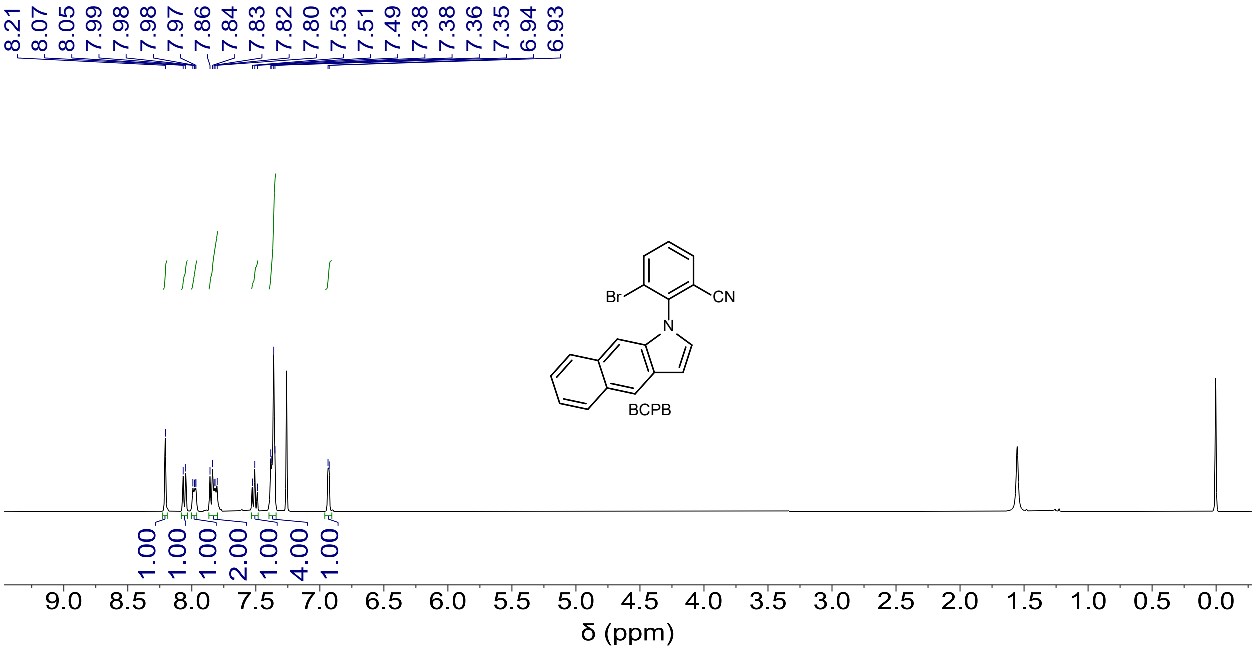

Supplementary Fig. 49 | ^{1}H NMR spectrum of BCPB (400 Mz, $CDCl_3$).

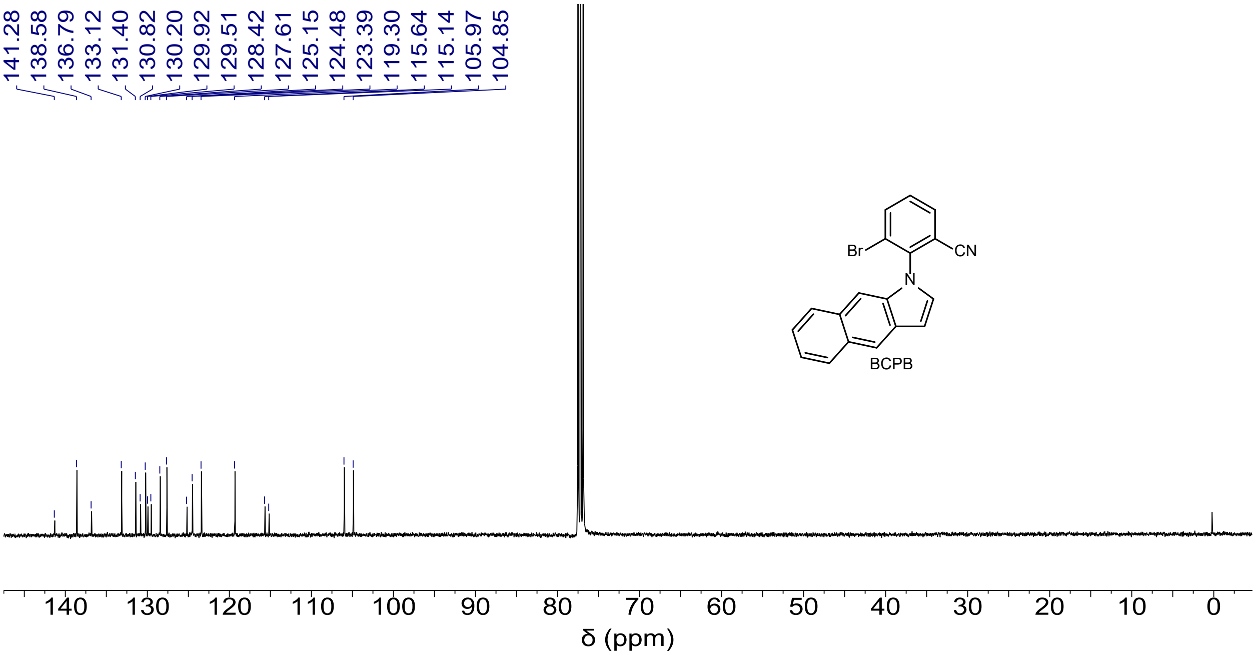

Supplementary Fig. 50 | ^{13}C NMR spectrum of BCPB (101 Mz, $CDCl_3$).

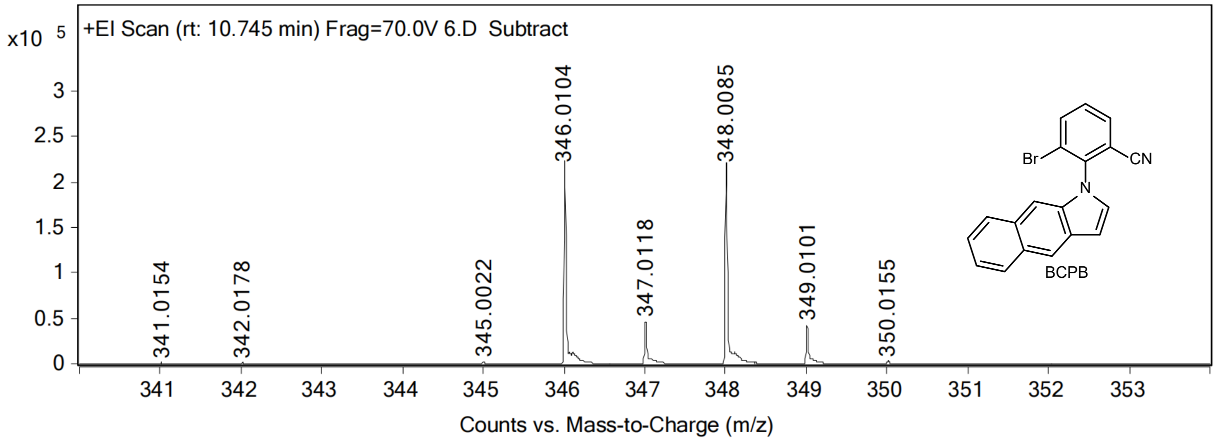

Supplementary Fig. 51 | EI-HRMS of BCPB.

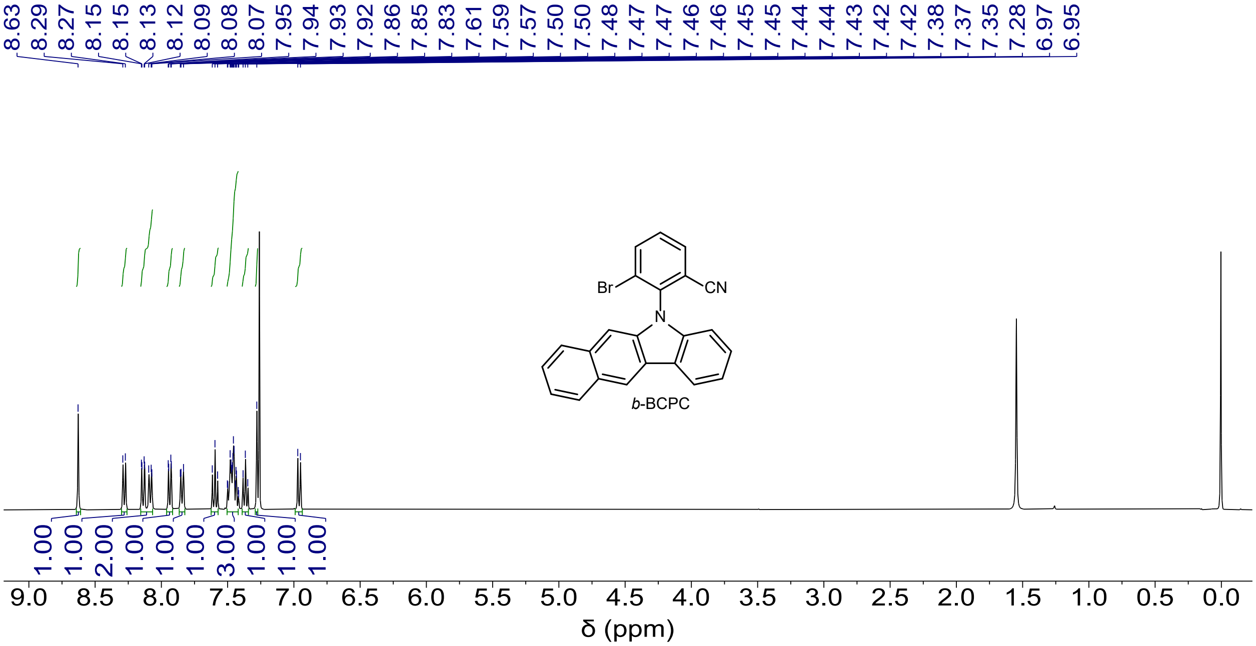

Supplementary Fig. 52 | ^{1}H NMR spectrum of *b*-BCPC (400 Mz, $CDCl_3$).

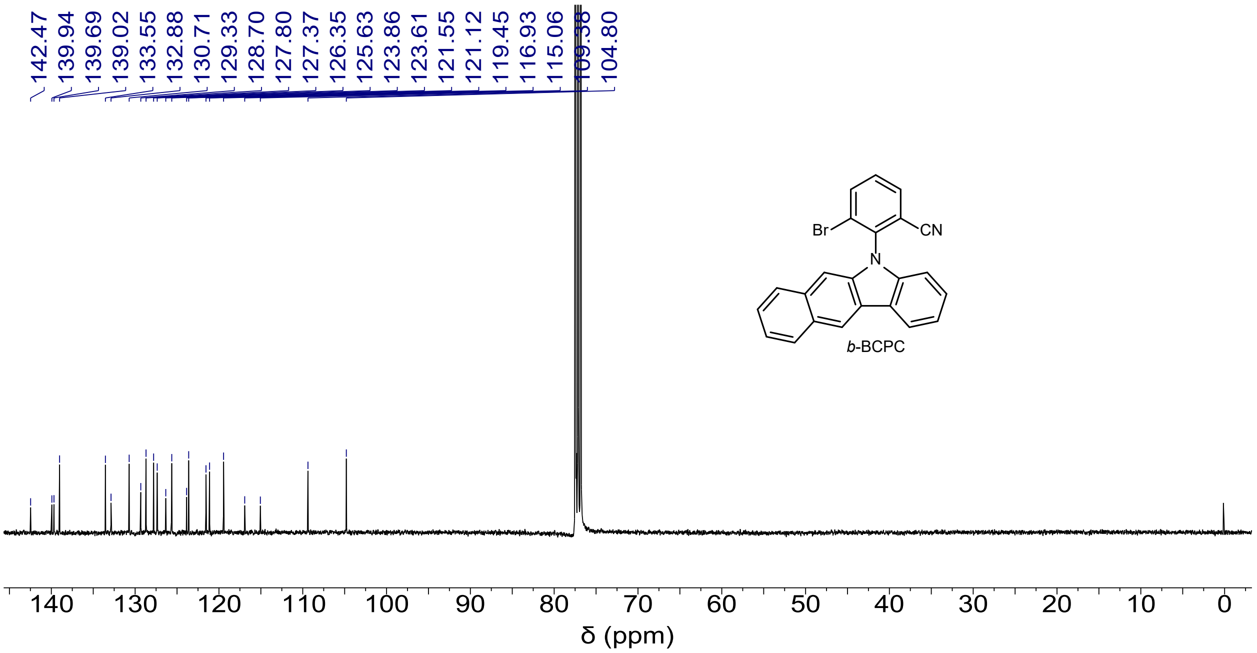

Supplementary Fig. 53 | ^{13}C NMR spectrum of *b*-BCPC (101 Mz, $CDCl_3$).

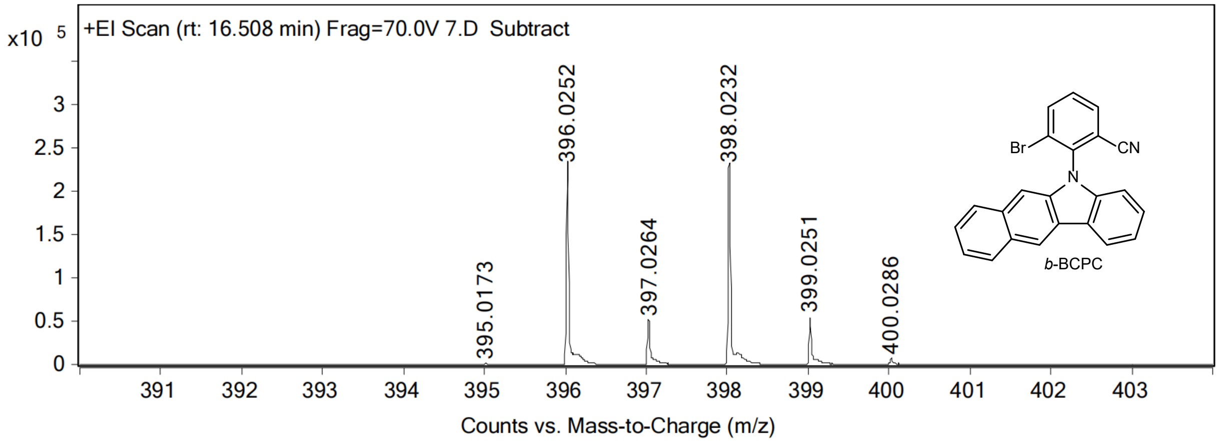

Supplementary Fig. 54 | EI-HRMS of *b*-BCPC.

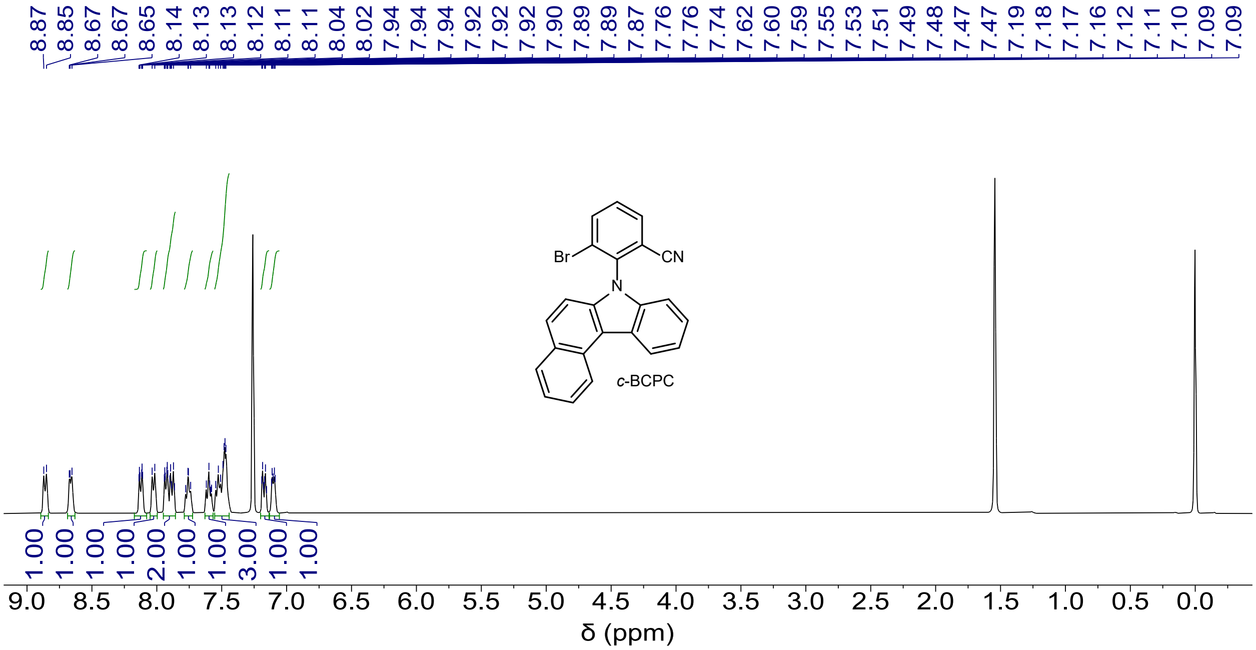

Supplementary Fig. 55 | ^{1}H NMR spectrum of *c*-BCPC (400 Mz, $CDCl_3$).

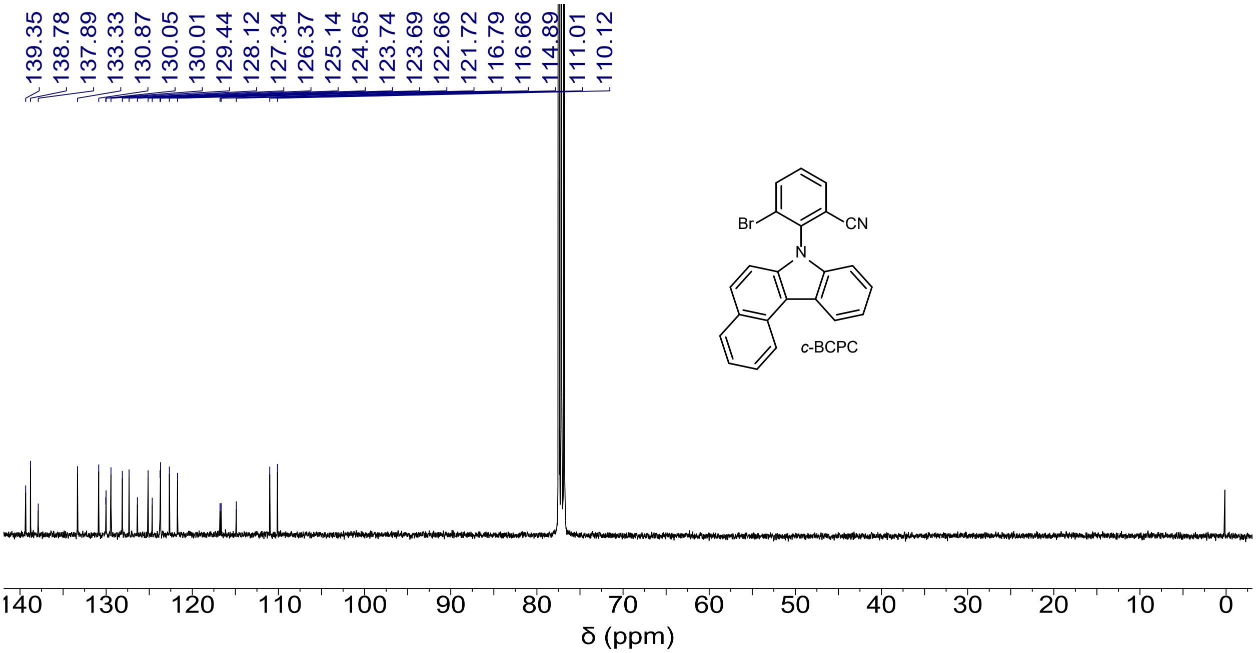

Supplementary Fig. 56 | ^{13}C NMR spectrum of *c*-BCPC (101 Mz, $CDCl_3$).

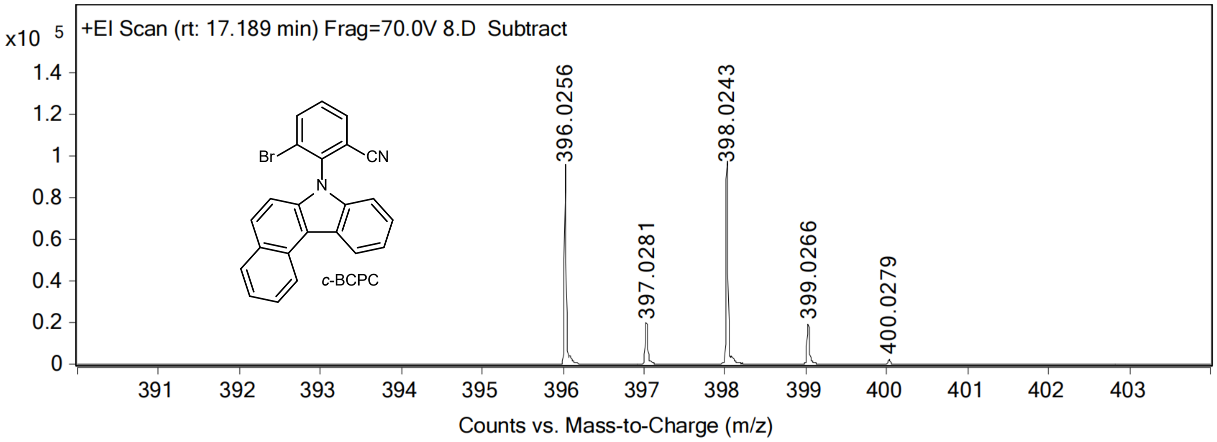

Supplementary Fig. 57 | EI-HRMS of *c*-BCPC.

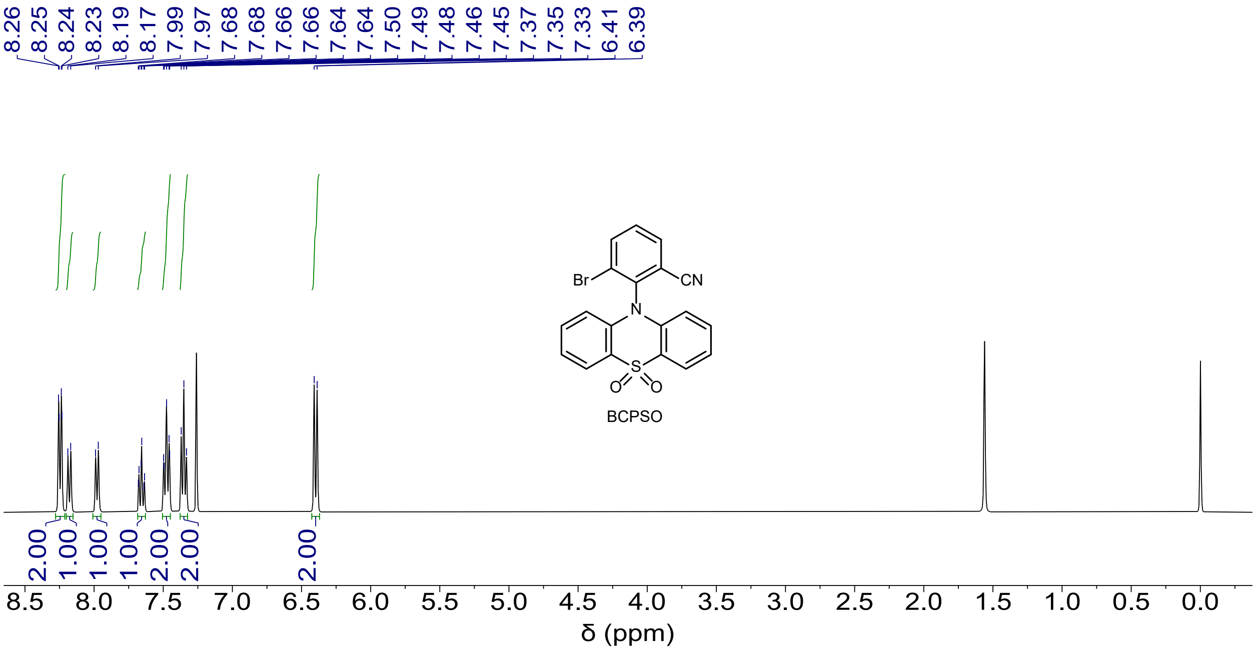

Supplementary Fig. 58 | ^{1}H NMR spectrum of BCPSO (400 Mz, $CDCl_3$).

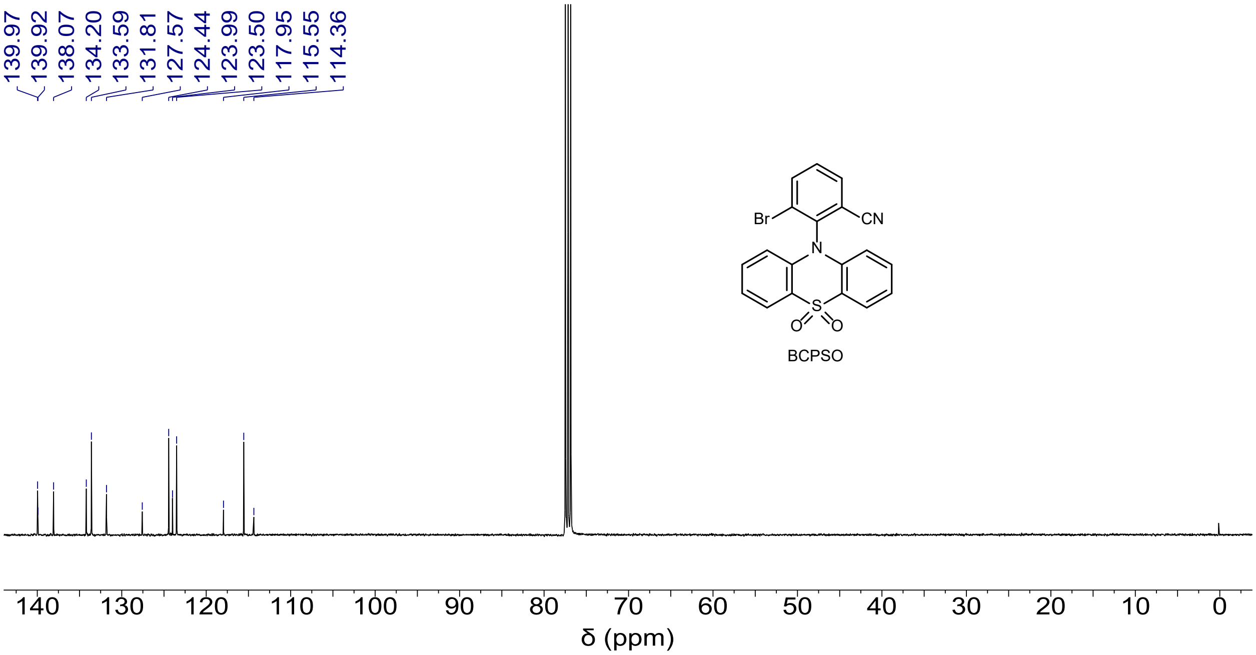

Supplementary Fig. 59 | ^{13}C NMR spectrum of BCPSO (101 Mz, $CDCl_3$).

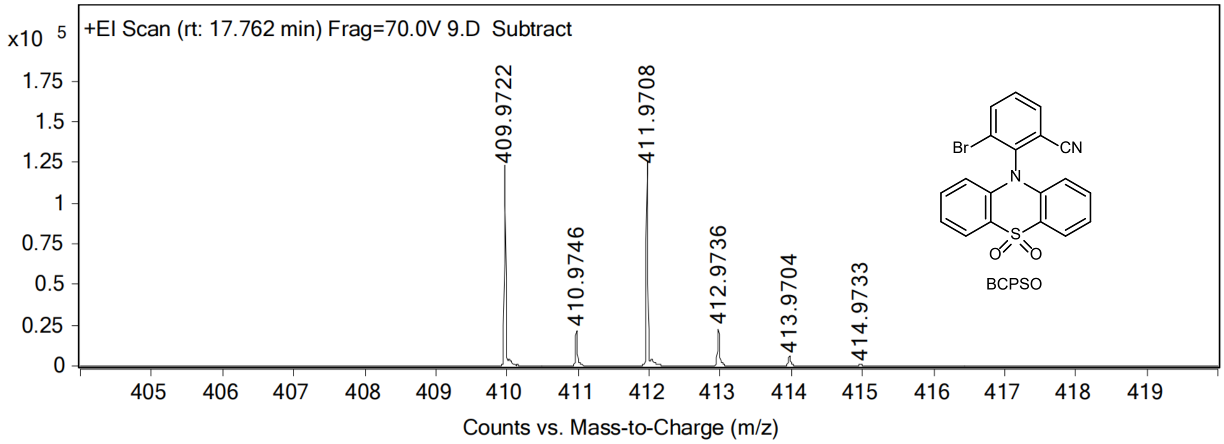

Supplementary Fig. 60 | EI-HRMS of BCPSO.

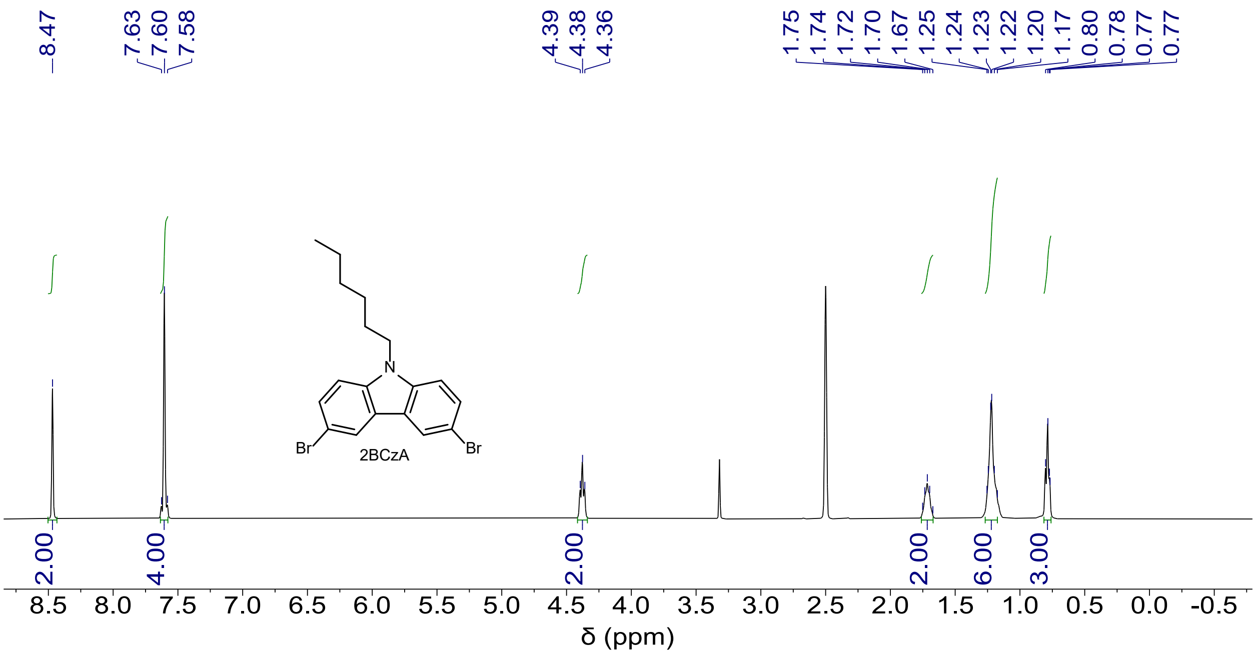

Supplementary Fig. 61 | ^{1}H NMR spectrum of 2BCzA (400 Mz, DMSO-d_6).

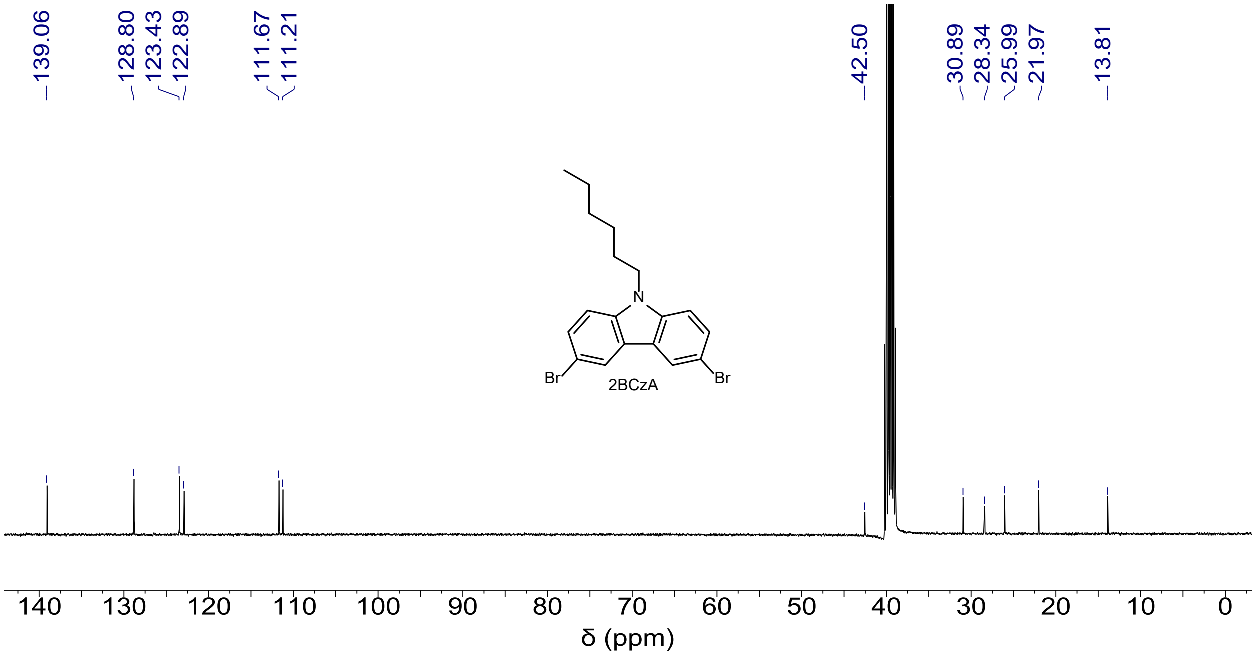

Supplementary Fig. 62 | ^{13}C NMR spectrum of 2BCzA (101 MHz, DMSO-d_6).

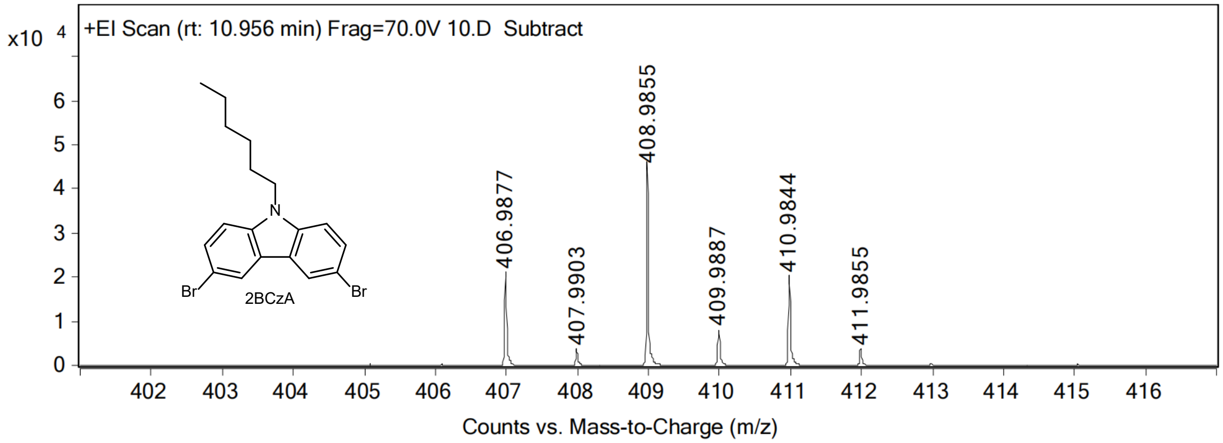

Supplementary Fig. 63 | EI-HRMS of 2BCzA.

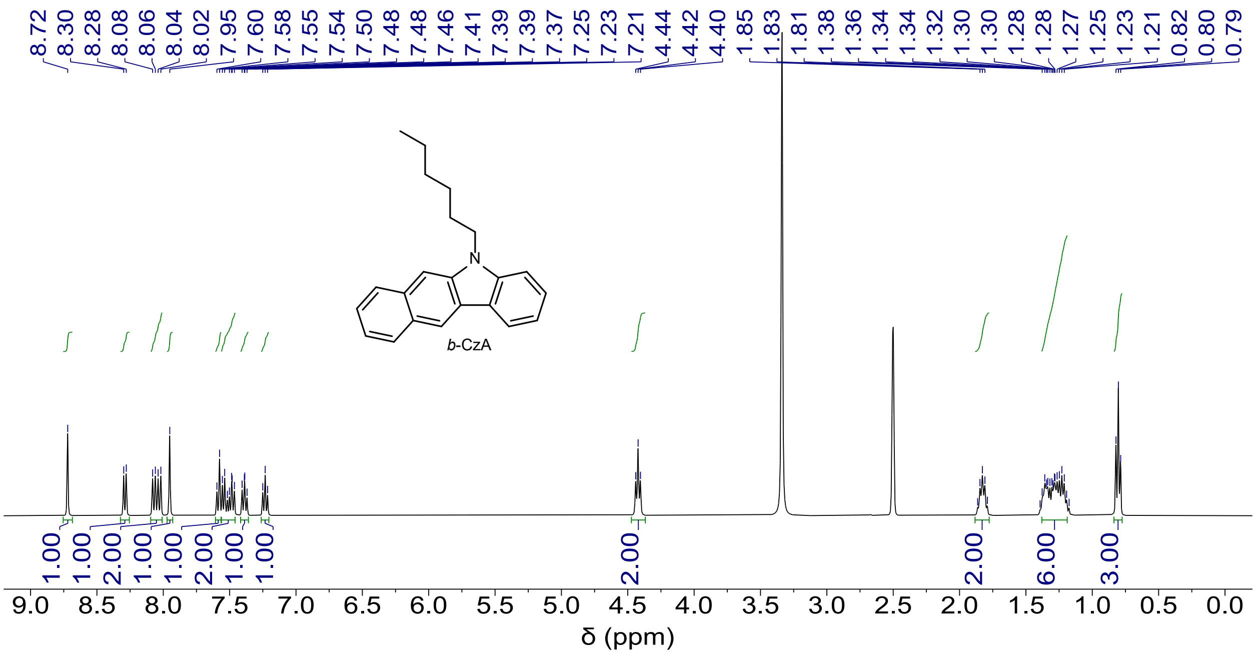

Supplementary Fig. 64 | ^{1}H NMR spectrum of *b*-CzA (400 MHz, DMSO-d_6).

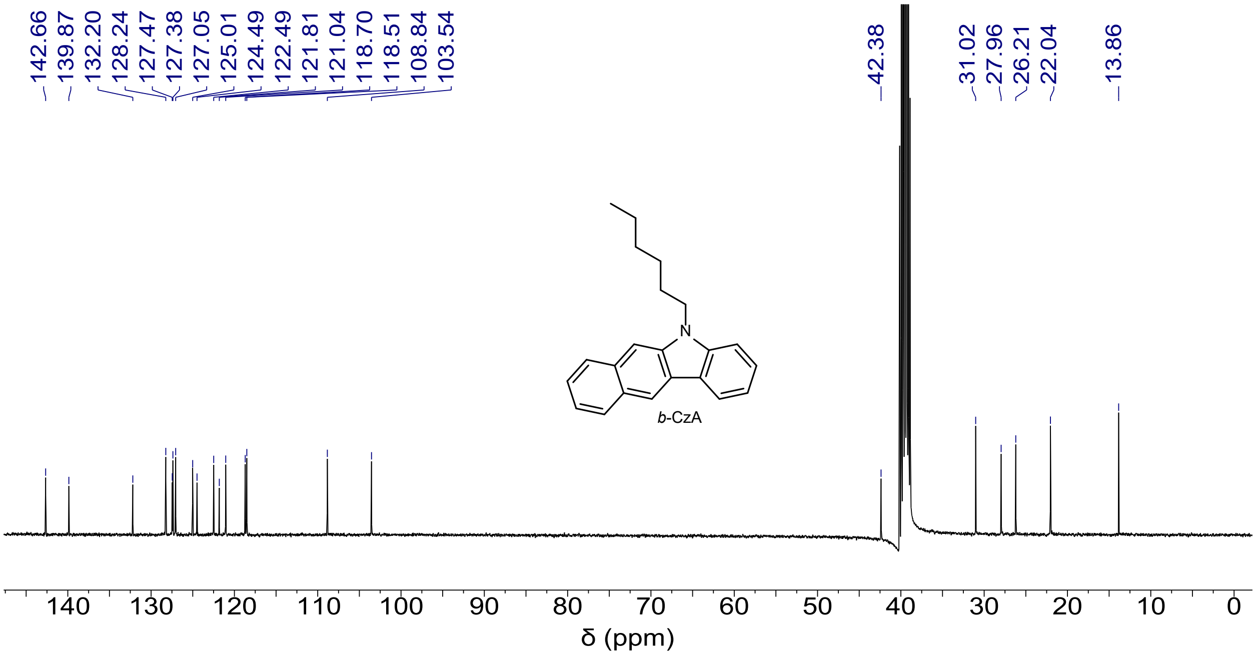

Supplementary Fig. 65 | ^{13}C NMR spectrum of *b*-CzA (101 MHz, DMSO-d_6).

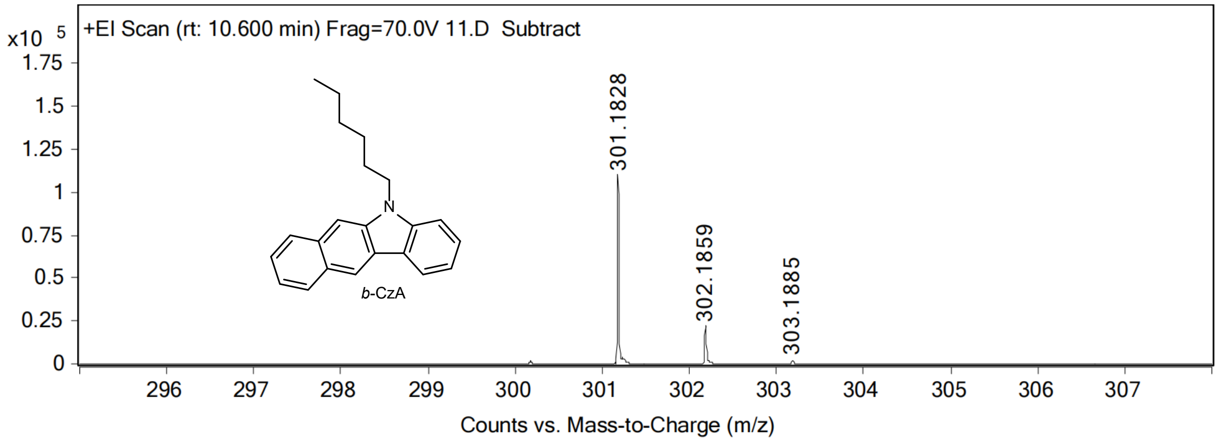

Supplementary Fig. 66 | EI-HRMS of *b*-CzA.

Supplementary Tables

Supplementary Table 1. Photophysical properties of BCPC&BCPB with varying doping ratios.

Samples	λ_{PL} (nm)	λ_{Phos} (nm)	τ_{Phos} (ms)	Φ_{PL} (%)	R_{Phos} (%)	Φ_{Phos} (%)
0.1%	434, 564, 611, 665	565, 615, 671	112.9	1.46	91.4	1.33
0.5%	437, 564, 611, 665	565, 615, 671	109.6	1.76	91.7	1.61
1%	445, 564, 611, 665	565, 614, 672	114.2	2.09	91.8	1.92
5%	447, 564, 612, 665	565, 615, 671	111.6	1.09	64.2	0.70
10%	497, 564, 611, 665	565, 615, 670	101.4	0.88	66.4	0.58

Supplementary Table 2. Photophysical properties of the host and doping systems at room temperature.

Samples	λ_{PL} (nm)	λ_{Phos} (nm)	λ_{ML} (nm)	λ_{pML} (nm)	τ_{Phos} (ms)	Φ_{PL} (%)	R_{Phos} (%)	Φ_{Phos} (%)
PC	381	-	383	inactive	-	7.27	-	-
PC&PB	400	399, 565, 618, 677	401	inactive	278.3 (565 nm)	7.87	< 1.0	< 0.08
BPC	421	-	423	inactive	-	0.88	-	-
BPC&BPB	403, 567, 612, 675	567, 613, 673	405	inactive	171.4 (567 nm)	0.99	18.7	0.19
BCPC	414	-	426	inactive	-	0.64	-	-
BCPC&BCPB	430, 565, 613, 674	565, 613, 673	565, 613, 674	565, 613, 674	114.2 (565 nm)	2.09	91.8	1.92
BCPC&*b*-BCPC	421, 541, 588, 641, 704	542, 590, 646, 708	543, 591, 646	543, 591, 646	233.7 (542 nm)	12.99	91.5	11.89
BCPC&*c*-BCPC	417, 497, 536, 579, 631	498, 537, 580, 630	499, 537, 581	499, 537, 581	18.8 (498 nm)	6.70	91.3	6.12
BCPSO&*b*-BCPC	419, 544, 592, 645, 708	546, 594, 649, 711	546, 594, 646	546, 594, 646	384.1 (546 nm)	7.60	95.4	7.25
BCPSO&*c*-BCPC	417, 494, 532, 573, 625	499, 537, 581, 629	495, 534, 581	495, 534, 581	319.4 (499 nm)	3.73	93.6	3.49

* Based on the phosphorescence band structure and delayed emission spectra, it can distinguish readily between fluorescence and phosphorescence bands in steady-state emission spectra. The ratios of phosphorescence components (R_{Phos}) in steady-state emission spectra were determined by calculating the areas of the separated fluorescence and phosphorescence bands and then dividing them by that of the steady-state emission band. Subsequently, the quantum yields of phosphorescence (Φ_{Phos}) for these doping materials were determined based on their total photoluminescence quantum yields (Φ_{PL}) and corresponding R_{Phos}.

Supplementary Table 3. TD-DFT calculation results of excited states of BCPC&BCPB optimized from BCPC single crystal.

Transition	E(eV)	D (Å)	S_r	t (Å)	Transition	E(eV)	D (Å)	S_r	t (Å)
$S_0 \rightarrow T_1$	2.25	0.29	0.86	-1.45	$S_0 \rightarrow S_1$	2.39	4.83	0.14	3.36
$S_0 \rightarrow T_2$	2.39	4.49	0.29	2.86	$S_0 \rightarrow S_2$	2.50	8.38	0.03	6.86
$S_0 \rightarrow T_3$	2.50	8.37	0.04	6.84	$S_0 \rightarrow S_3$	2.91	7.77	0.08	5.90
$S_0 \rightarrow T_4$	2.90	7.32	0.11	5.38	$S_0 \rightarrow S_4$	2.98	5.87	0.12	4.06
$S_0 \rightarrow T_5$	2.97	6.22	0.14	4.34	$S_0 \rightarrow S_5$	3.18	3.81	0.20	2.36
$S_0 \rightarrow T_6$	3.14	3.33	0.42	1.65					

For TD-DFT calculation results, D index is defined as the distance between the hole and the electron center of mass. S_r index is defined as the function that describes the overlap between electron and hole distributions, the larger the S_r index, the higher the degree of overlap between hole and electron. While t index describes the degree of separation between the hole and the electron, positive t index implies that the hole and electron are separated sufficiently due to charge transfer (CT). For local excitation or global excitation, the excited states

featured small D, large S_r, and obviously negative t. On the contrary, for CT excitation, they often show large D, small S_r, and positive t.

Supplementary Table 4. TD-DFT calculation results of excited states of BPC&BPB optimized from BPC single crystal.

Transition	E(eV)	D (Å)	S_r	t (Å)	Transition	E(eV)	D (Å)	S_r	t (Å)
$S_0 \rightarrow T_1$	2.25	0.14	0.87	-2.00	$S_0 \rightarrow S_1$	3.49	4.49	0.32	2.86
$S_0 \rightarrow T_2$	3.21	0.09	0.93	-1.69	$S_0 \rightarrow S_2$	3.56	0.88	0.82	-1.49
$S_0 \rightarrow T_3$	3.27	2.97	0.68	0.98	$S_0 \rightarrow S_3$	3.59	4.77	0.26	3.21
$S_0 \rightarrow T_4$	3.37	0.55	0.75	-1.14	$S_0 \rightarrow S_4$	3.72	6.10	0.06	4.81
$S_0 \rightarrow T_5$	3.51	0.21	0.84	-1.42	$S_0 \rightarrow S_5$	3.78	7.38	0.04	6.08
$S_0 \rightarrow T_8$	3.71	4.92	0.41	2.94	$S_0 \rightarrow S_7$	3.97	3.48	0.37	1.84

Supplementary Table 5. TD-DFT calculation results of excited states of PC&PB optimized from PC single crystal.

Transition	E(eV)	D (Å)	S_r	t (Å)	Transition	E(eV)	D (Å)	S_r	t (Å)
$S_0 \rightarrow T_1$	2.23	0.14	0.87	-1.95	$S_0 \rightarrow S_1$	3.51	1.07	0.81	-1.12
$S_0 \rightarrow T_2$	3.20	0.06	0.89	-1.60	$S_0 \rightarrow S_2$	3.70	6.38	0.07	4.98
$S_0 \rightarrow T_3$	3.36	0.49	0.76	-1.22	$S_0 \rightarrow S_3$	3.88	3.66	0.50	1.52
$S_0 \rightarrow T_4$	3.46	0.31	0.89	-1.82	$S_0 \rightarrow S_4$	3.93	4.63	0.28	7.70
$S_0 \rightarrow T_5$	3.64	1.17	0.86	-1.41	$S_0 \rightarrow S_5$	3.99	0.87	0.73	-0.86
$S_0 \rightarrow T_6$	3.70	5.97	0.17	4.10					

Supplementary Table 6. Crystal data and structure refinement for single crystal of PC.

**Formula**	$C_{18}H_{13}N$	**ρ_{calc}/g cm^{-3}**	1.253
**Formula weight**	243.29 g/mol	**Z**	16
**Temperature/K**	100(2)	**Absorption coefficient**	0.558
**Crystal system**	orthorhombic	**F(000)**	2048
**Space group**	*Fdd2*	**Crystal size/mm³**	0.138 × 0.176 × 0.179
**a/Å**	12.6575(3)	**Wavelength**	1.54178 Å
**b/Å**	37.9423(8)	**Reflections collected**	16631
**c/Å**	10.7458(2)	**Data/restraints/parameters**	2494 / 1 / 173
**α/°**	90	**Independent reflections**	2494 [R(int) = 0.0189]
**β/°**	90	**Goodness-of-fit on F^2**	1.061
**γ/°**	90	**R_1,[a] wR_2[b] [$I \geq 2\sigma(I)$]**	R_1 = 0.0240, wR_2 = 0.0598
**Volume/Å³**	5160.72(19)	**R_1, wR_2 [all data]**	R_1 = 0.0240, wR_2 = 0.0598

Supplementary Table 7. Crystal data and structure refinement for single crystal of BPC.

**Formula**	$C_{18}H_{12}BrN$	**ρ_{calc}/g cm^{-3}**	1.499
**Formula weight**	322.20 g/mol	**Z**	8
**Temperature/K**	100(2)	**Absorption coefficient**	2.868 mm^{-1}
**Crystal system**	orthorhombic	**F(000)**	1296
**Space group**	*Iba*2	**Crystal size/mm^3**	0.110 × 0.156 × 0.240
**a/Å**	14.7598(11)	**Wavelength**	0.71073 Å
**b/Å**	24.6995(17)	**Reflections collected**	42808
**c/Å**	7.8309(6)	**Data/restraints/parameters**	3990 / 1 / 181
**α/°**	90	**Independent reflections**	3990 [R(int) = 0.0508]
**β/°**	90	**Goodness-of-fit on F^2**	1.076
**γ/°**	90	**R_1,[a] wR_2[b] [$I\geq2\sigma(I)$]**	R_1 = 0.0207, wR_2 = 0.0488
**Volume/Å^3**	2854.8(4)	**R_1, wR_2 [all data]**	R_1 = 0.0238, wR_2 = 0.0498

Supplementary Table 8. Crystal data and structure refinement for single crystal of BCPC.

**Formula**	$C_{19}H_{11}BrN_2$	**ρ_{calc}/g cm^{-3}**	1.530
**Formula weight**	347.21 g/mol	**Z**	4
**Temperature/K**	105(2)	**Absorption coefficient**	2.724 mm^{-1}
**Crystal system**	monoclinic	**F(000)**	696
**Space group**	$P2_1$	**Crystal size/mm^3**	0.132 × 0.198 × 0.215
**a/Å**	8.2657(7)	**Wavelength**	0.71073 Å
**b/Å**	12.4850(11)	**Reflections collected**	33977
**c/Å**	14.6196(12)	**Data/restraints/parameters**	8424 / 1 / 397
**α/°**	90	**Independent reflections**	8424 [R(int) = 0.0484]
**β/°**	92.377(3)	**Goodness-of-fit on F^2**	1.038
**γ/°**	90	**R_1,[a] wR_2[b] [$I\geq2\sigma(I)$]**	R_1 = 0.0329, wR_2 = 0.0763
**Volume/Å^3**	1507.4(2)	**R_1, wR_2 [all data]**	R_1 = 0.0382, wR_2 = 0.0787

Supplementary Table 9. Crystal data and structure refinement for single crystal of BCPSO.

**Formula**	$C_{19}H_{11}BrN_2O_2S$	**ρ_{calc}/g cm^{-3}**	1.672
**Formula weight**	411.27 g/mol	**Z**	8
**Temperature/K**	100(2)	**Absorption coefficient**	2.659 mm^{-1}
**Crystal system**	monoclinic	**F(000)**	1648
**Space group**	$P2_1$	**Crystal size/mm^3**	0.190 × 0.236 × 0.274

**a/Å**	16.6718(7)	**Wavelength**	0.71073 Å
**b/Å**	12.2031(5)	**Reflections collected**	91443
**c/Å**	16.8521(7)	**Data/restraints/parameters**	18228 / 157 / 1013
α/°	90	**Independent reflections**	18228 [R(int) = 0.0553]
β/°	107.5780(10)	**Goodness-of-fit on F^2**	1.053
γ/°	90	R_1,[a] wR_2[b] [$I \geq 2\sigma(I)$]	$R_1 = 0.0313$, $wR_2 = 0.0712$
**Volume/Å^3**	3268.4(2)	R_1, wR_2 **[all data]**	$R_1 = 0.0363$, $wR_2 = 0.0725$

Supplementary References

1. Zhang X, et al. A Class of Organic Units Featuring Matrix-Controlled Color-Tunable Ultralong Organic Room Temperature Phosphorescence. *Adv. Sci.* **10**, 2206482 (2023).
2. Tamai Y, Shirouchi R, Saito T, Kohzuki K, Natsuda S-i. Role of the energy offset in the charge photogeneration and voltage loss of nonfullerene acceptor-based organic solar cells. *J. Mater. Chem. A* **11**, 17581-17593 (2023).
3. Jasiūnas R, Zhang H, Gelžinis A, Chmeliov J, Franckevičius M, Gao F, Gulbinas V. Interplay between charge separation and hole back transfer determines the efficiency of non-fullerene organic solar cells with low energy level offset. *Org. Electron.* **108**, 106601 (2022).
