# Supplementary material for: Isostructural doping for organic persistent mechanoluminescence
Source: Nat Commun. 2024 Apr 30;15:3668. doi: 10.1038/s41467-024-47962-6 (PMC11063035; doi:10.1038/s41467-024-47962-6)
Supplement: Supplementary file 3 — Description of Additional Supplementary Files [file 41467_2024_47962_MOESM3_ESM.pdf]

Description of Additional Supplementary Files

File Name: Supplementary Data 1

Description: The atomic coordinates of the computational models (PC, PC&PB, BPC, BPC&BPB, BCPC, BCPC&BCPB).
